# Recurrent mutations in protein tyrosine phosphatase receptor-type kappa in depressed-type colorectal carcinomas

**DOI:** 10.1093/jb/mvag024

**Published:** 2026-03-12

**Authors:** Masayuki Tojo, Keiko Shinjo, Fumiharu Ohka, Keisuke Katsushima, Akira Hatanaka, Norihisa Ichimura, Hiromu Suzuki, Hisako Nozawa, Hitoshi Yoshida, Kazuo Konishi, Yutaka Kondo

**Affiliations:** Division of Gastroenterology, Showa Medical University School of Medicine, 1-5-8 Hatanodai, Shinagawa-ku, Tokyo, 142-8555, Japan; Division of Cancer Biology, Nagoya University Graduate School of Medicine, 65 Tsurumai-cho, Showa-ku, Nagoya 466-8550, Japan; Division of Cancer Biology, Nagoya University Graduate School of Medicine, 65 Tsurumai-cho, Showa-ku, Nagoya 466-8550, Japan; Department of Neurosurgery, Nagoya University Graduate School of Medicine, 65 Tsurumai-cho, Showa-ku, Nagoya 466-8550, Japan; Division of Cancer Biology, Nagoya University Graduate School of Medicine, 65 Tsurumai-cho, Showa-ku, Nagoya 466-8550, Japan; Division of Cancer Biology, Nagoya University Graduate School of Medicine, 65 Tsurumai-cho, Showa-ku, Nagoya 466-8550, Japan; Division of Cancer Biology, Nagoya University Graduate School of Medicine, 65 Tsurumai-cho, Showa-ku, Nagoya 466-8550, Japan; Department of Oral and Maxillofacial Surgery, Komaki City Hospital, 1-20 Jobushin, Komaki City, 485-8520, Japan; Division of Molecular Biology, Department of Biochemistry, Sapporo Medical University, 17, Minami-1-jonishi, Chuo-ku, Sapporo City, 060-8556, Japan; Division of Gastroenterology, Showa Medical University School of Medicine, 1-5-8 Hatanodai, Shinagawa-ku, Tokyo, 142-8555, Japan; Division of Gastroenterology, Showa Medical University School of Medicine, 1-5-8 Hatanodai, Shinagawa-ku, Tokyo, 142-8555, Japan; Division of Gastroenterology, Showa Medical University School of Medicine, 1-5-8 Hatanodai, Shinagawa-ku, Tokyo, 142-8555, Japan; Division of Cancer Biology, Nagoya University Graduate School of Medicine, 65 Tsurumai-cho, Showa-ku, Nagoya 466-8550, Japan

**Keywords:** colorectal cancer, CpG island methylator phenotype, depressed-type colorectal cancer, ITGB4, PTPRK mutation

## Abstract

Genetic and epigenetic alterations in colorectal cancer (CRC) have been extensively investigated. Small depressed-type colorectal tumours (DCs) constitute a highly invasive CRC subtype. We characterized the mutational profiles of 22 DC samples, seven of which were subjected to whole exome sequencing. This revealed frequent mutations (7 of 22 cases, 31.8%) in *PTPRK*, with most of these mutations located in the D1 domain-encoding region. To further quantify the incidence of *PTPRK* mutations, we analysed 567 CRCs cases from two public databases (The Cancer Genome Atlas and the Catalogue of Somatic Mutations in Cancer). Mutations were more frequently observed in proximal colon tumours and were highly associated with the CpG island methylator phenotype-positive (CIMP-P) subtype (76.2%) and microsatellite instability (MSI; 66.7%). PTPRK mutant proteins (particularly those with D1 domain) retained the ability to bind integrin beta-4 (ITGB4). In addition, CRC cells expressing PTPRK mutants exhibited increased ITGB4 phosphorylation, suggesting that the mutations impair the phosphatase activity of PTPRK. Consistent with this, tumours with PTPRK mutations proliferated significantly more rapidly than their wild-type counterparts *in vivo*. These findings suggest that *PTPRK* mutations contribute to DC development by dysregulating phosphorylation-mediated signalling pathways.

## Graphical Abstract

**Graphical Abstract:**
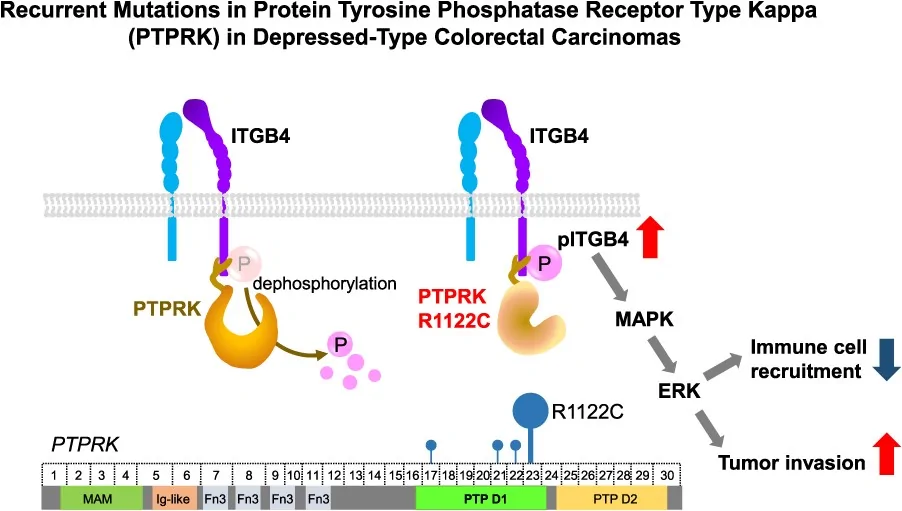

## Abbreviations

CIMP: CpG island methylator phenotype
DC: depressed-type colorectal tumour
ITGB4: interacts with integrin beta 4
MSI: microsatellite instability
PTPRK: protein tyrosine phosphatase receptor type K

Colorectal cancer (CRC) is one of the leading causes of cancer-related deaths worldwide *(**1**)*. Recent comprehensive genome-wide analyses show that molecular profiles are clinically relevant determinants of CRC characteristics; specific genetic alterations are associated with patient survival, disease recurrence and therapeutic responses *(**2–4**)*.

As genetic alterations can inform therapeutic decisions and predict clinical outcomes in CRC, they have been exploited to develop numerous targeted treatment options *(**5**)*. However, the treatment of unresectable, advanced or recurrent CRC remains a significant challenge and is often associated with a poor prognosis, underscoring the urgent need for novel therapeutic strategies. To identify effective therapeutic targets for CRC, a comprehensive understanding of its carcinogenic processes is essential.

The signalling aberrations involved in the formation of colorectal polyps; such as adenomas and serrated lesions differ from those associated with colorectal carcinogenesis. Moreover, the molecular phenotype of CRC varies depending on the tumour location, with factors such as the CpG island methylator phenotype (CIMP), microsatellite instability (MSI) and specific gene mutations playing key roles *(**2**,*  *6**,*  *7**)*. Furthermore, studies using CRC development models—validated through multi-region tumour analysis—have shown that CRCs typically harbour uniform driver gene mutations within individual tumours. However, neutral evolution contributes to the accumulation of heterogeneous mutations. In contrast, precancerous colorectal lesions are characterized by heterogeneous driver gene mutations within a single tumour *(**8**,*  *9**)*.

CRC can be classified based on its macroscopic morphological characteristics *(**10**)*. Among these, cancers with flat or depressed areas—referred to as depressed-type cancers (DCs; Supplementary Fig. S1)—tend to exhibit a high rate of submucosal invasion despite their small size *(**11–13**)*. In addition, the depth of submucosal invasion correlates with the risk of lymph node metastasis *(**14**)*.

DCs represent early clonal populations that have acquired malignant properties, and are thought to harbour relatively few genetic mutations through neutral evolution. Therefore, they are ideal tumour subtype for identifying genetic alterations strongly associated with early stage of CRC development *(**15**)*. Although APC and TP53 mutations are frequently reported in DCs, the identity of other molecular drivers is unclear *(**15**,*  *16**)*. Further elucidation of the molecular features of DCs may facilitate development of novel CRC therapeutic strategies.

In this study, we performed mutational analysis in DCs and found recurrent mutations in the protein tyrosine phosphatase receptor type K (*PTPRK*) gene. Analysis of public databases revealed that *PTPRK* mutations are more frequently observed in proximal colon cancer and are associated with poor prognosis. Furthermore, we found that PTPRK directly interacts with integrin beta 4 (ITGB4), and that mutations in PTPRK impair its phosphatase function, resulting in increased ITGB4 phosphorylation and promoted tumour proliferation *in vivo*. Collectively, these findings suggest that PTPRK mutations may drive the development of the DC subset of CRC through enhanced ITGB4 tyrosine phosphorylation.

## Materials and Methods

### CRC clinical samples and classification of CRCs

In this study, DC was defined as a lesion exhibiting a depressed morphology, with or without a flat elevated component (Supplementary Fig. S1), and characterized by tumour cell invasion beyond the submucosal layer. The macroscopic classification of CRC was performed according to the Paris endoscopic classification system *(**10**)*.

A total of 22 DCs samples and 8 matched normal colonic epithelial tissues were obtained from 22 patients who underwent surgical treatment or endoscopic resection at Showa University Hospital and Akita Red Cross Hospital, Japan. Sample collection was conducted between 2010 and 2015 after following approval from the institutional review board (approval number: 129), and written informed consent was obtained from all participants prior to sample acquisition.

### Whole exome sequencing

Genomic DNA extracted from seven paired samples of DCs and their corresponding normal tissues was subjected to whole exome sequencing (WES). Exome capture was performed using the SureSelect Human Exon v5 (Agilent Technologies, Santa Clara, CA, USA), following the Illumina Multiplex Sequencing Ver1.5 protocol (Illumina, San Diego, CA, USA). Briefly, 3 μg of genomic DNA from each sample was sonicated using an S220 sonicator (Covaris, Inc., Woburn, MA, USA) to generate fragments ranging from 150 to 200 base pairs (bp). After ligation of adapter sequences to both ends of the DNA fragments, fragment size and concentration were assessed using Bioanalyzer (Agilent Technologies). Hybridization and exon capture were then performed, targeting 50 Mb of exonic regions corresponding to 21,522 genes. Barcode adaptor sequences were subsequently ligated to each sample, and sequencing was conducted on the Illumina MiSeq system using the Reagent Kit v2 (Illumina). Sequences contigs were aligned to the human reference genome (hg19), and variant calling was performed to identify single nucleotide polymorphisms (SNPs), insertions and deletions. Common SNPs in dbSNP built 137 and HapMap data, as well as germline variants identified in matched normal tissue, were filtered out. Next-generation sequencing (NGS) data were analysed using CLC Genomics Workbench v6.5.1 (Qiagen, Hilden, Germany). For variant detection, thresholds for minimum coverage and minimum variant frequency were set at 10x and 15%, respectively, for both tumour and matched normal tissue samples.

### Mutation analysis

Exons 16–24 of the *PTPRK* gene were amplified by PCR and analysed by direct sequencing in 15 DC samples. Validation of *PTPRK* mutations identified through WES was performed using pyrosequencing. Mutations in codons 12 and 13 of the *KRAS* gene and codon 600 of the *BRAF* gene were also analysed by pyrosequencing, as previously described *(**17**,*  *18**)*. *TP53* mutations (exons 5–8) were assessed by direct sequencing *(**19**)*. The primers used for these assays are listed in Supplementary Table S1.

### MSI analysis

MSI status was determined as previously described *(**20**)*. Briefly, DNA samples from tumour tissues and adjacent normal tissues were amplified, and five MSI markers (BAT25, BAT26, D2S123, D5S346 and D17S250) were evaluated. A tumour sample was classified as MSI-positive if one or more of the five markers exhibited instability.

### DNA methylation analysis

CpG island methylator phenotype (CIMP) status was assessed using five markers (*MINT1, MINT2, MINT31, P16 and hMLH1*) by pyrosequencing, as previously reported *(**21**)*. The primers used for this analysis are listed in Supplementary Table S1.

### Reverse transcription-quantitative PCR

Total RNA was extracted using the FastGene™ RNA Basic Kit (NIPPON Genetics, Tokyo, Japan). RNA concentration was measured with a NanoDrop ND-1000 spectrophotometer (Thermo Fisher Scientific, Waltham, MA, USA), and 200 ng of total RNA was used for reverse transcription with the PrimeScript™ RT Reagent Kit with gDNA Eraser (Takara Bio, Shiga, Japan). The resulting cDNA was subjected to quantitative PCR (qPCR) using the KAPA SYBR Fast qPCR kit (NIPPON Genetics) on a CFX Connect Real-Time PCR System (BioRad, Hercules, CA, USA). Expression levels of target genes were normalized to β-actin as an internal control. Primer sequences used in this analysis are listed in Supplementary Table S1.

### Public database analysis

Publicly accessible clinical datasets of CRCs, including information on genetic alterations, CIMP status and MSI status, were obtained from The Cancer Genome Atlas (TCGA) and the Catalogue of Somatic Mutations in Cancer (COSMIC) database. Survival analysis for *PTPRK*-mutant and wild-type (WT) cases was performed using CRC data (TCGA, provisional) via the cBioPortal web tool (https://www.cbioportal.org). Overall survival was analysed using Kaplan–Meier survival curves, and statistical significance was assessed by the log-rank test.

### Cell lines

CRC cell lines—HCT116, HT29, RKO, Caco-2, SW48 and LS411N—were obtained from the American Type Culture Collection (ATCC, Manassas, VA, USA). HCT15 cells were obtained from the Cell Resource Centre for Biomedical Research, Institute of Development, Aging and Cancer Tohoku University, Japan. HCT116, HT29, RKO and Caco-2 cells were maintained in DMEM medium (FUJIFILM Wako Pure Chemical Corp., Osaka, Japan), while SW48, LS411N and HCT15 cells were cultured in RPMI-1640 medium (FUJIFILM Wako Pure Chemical Corp.). All cells were grown in medium supplemented with 10% foetal bovine serum (Thermo Fisher Scientific, Waltham, MA, USA) and 1% penicillin/streptomycin (FUJIFILM Wako Pure Chemical Corp.) at 37°C in a humidified incubator with 5% CO_2_. Transfection was performed using Lipofectamine 3000 (Thermo Fisher Scientific) according to the manufacture’s protocol. Cells were harvested 72 hours post-transfection for further analysis.

### Construction of PTPRK mutant CRC cells

HCT116 and HT29 cells harbouring the PTPRK R1122C missense mutation (designated as HCT116^R1122C^ and HT29^R1122C^) were generated using the Clustered Regularly Interspaced Short Palindromic Repeat (CRISPR)-CRISPR-associated protein (Cas) system. A Cas9 D10A mutant nickase expression vector (CP-C9NI-02), a pair of single guide RNA (sgRNA) vectors containing guide sequences 5′- ATTAATACGCCGAGATCTTA-3′ and 5′-GTATGTAGATTTACCTGTAG-3′, and a homology-directed repair donor vector encoding the PTPRK R1122C mutation were designed and purchased from GeneCopoeia (Rockville, MD, USA). These vectors were co-transfected into cells using Lipofectamine 3000 (Thermo Fisher Scientific) according to the manufacture’s protocol. Transfected cells were selected with 1 μg/ml puromycin. Successful introduction of the *PTPRK* mutation was confirmed by PCR using specific primers targeting the mutated genomic sequence, followed by sequencing analysis. The primers used in this analysis are listed in Supplementary Table S1.

### Vector construction

The full-length *PTPRK* cDNA sequence was amplified from the BC144512 vector (transOMIC Technologies, Huntsville, AL, USA) using primers containing 5′ overhang sequences for the restriction enzyme sites HindIII and XhoI (Primer A and B, respectively). The PCR product was subcloned into the HindIII/XhoI sites of the pcDNA3.0/3 × FLAG vector [pcDNA3.0 (Thermo Fisher Scientific) containing the coding sequence for 3 × FLAG], generating the pcDNA3.0/3 × FLAG-*PTPRK* WT vector. The *PTPRK* mutant vector (pcDNA3.0/3xFLAG-*PTPRK* R1122C) was constructed using the QuickChange Site-Directed Mutagenesis Kit (Agilent Technologies). The pcDNA3.0/3 × FLAG-*PTPRK* WT vector was used as a template, and site-directed mutagenesis was performed using Primers C and D. The sequences of Primers A-D are listed in Supplementary Table S1. FLAG-HA-GFP vector was a gift from Wade Harper (Addgene plasmid # 22612; http://n2t.net/addgene:22612; RRID:Addgene_22,612) *(**22**)*.

### Global protein phosphorylation analysis

Phospho Explorer antibody microarray analysis was performed using the PEX100 microarray chip, which contains 1318 site-specific and phospho-specific antibodies (Full Moon Biosystems, Sunnyvale, CA, USA). Cell lysates from HCT116 and HCT116^R1122C^ cells were applied to the microarray and processed according to the manufacturer’s instructions (https://www.fullmoonbio.com/product/phospho-explorer-antibody-array/). Processed slides were scanned using a GenePix 4400A scanner (Molecular Devices, Sunnyvale, CA, USA), and the resulting images were analysed with Array-Pro Analyzer Ver.4.5 (Media Cybernetics, Silver Spring, MD, USA). The average fluorescence signal intensity of each replicate antibody spot was normalized to the median signal intensity of all antibody spots. Finally, the log_2_ ratio of normalized phosphoprotein signal intensity to non-phosphoprotein signal intensity was calculated.

### Western blot analysis

Cells were washed with ice-cold PBS and lysed in RIPA buffer (FUJIFILM Wako Pure Chemical Corp.) supplemented with protease and phosphatase inhibitor cocktails (Roche, Mannheim, Germany). Lysates were mixed with 4x Laemmli buffer (BioRad) and boiled at 95 °C for 5 minutes. Total protein samples (20 μg) were separated by sodium dodecyl sulphate-polyacrylamide gel electrophoresis (SDS-PAGE) and transferred onto polyvinylidene fluoride (PVDF) membrane. Immunoblotting was performed using the following primary antibodies: anti-PTPRK (28151-1-AP, 1:1000, Proteintech, Tokyo, Japan), anti-ITGB4 Y1510 (ab192805, 1:500, Abcam, Cambridge, MA, USA), anti-ITGB4 (21738-1-AP, 1:1000, Proteintech) anti-EGFR Y1092 (#2234, 1:1000, Cell Signalling Technology, Danvers, MA, USA), anti-EGFR (#4267, 1:1000, Cell Signalling Technology), anti-STAT3 Y705 (#9145, 1:1000, Cell Signalling Technology), anti-STAT3 (#9139, 1:1000, Cell Signalling Technology) and anti-β-actin (010-27841, 1:1000, FUJIFILM Wako Pure Chemical Corp.). Membranes were then incubated with HRP-linked secondary antibodies: anti-rabbit IgG (#7074, 1:1000, Cell Signalling Technology), or anti-mouse IgG (#7076, 1:1000, Cell Signalling Technology). Protein bands were visualized using ECL Prime Western Blotting Detection Reagent (Cytiva, Marlborough, MA, USA) and detected with the ImageQuant LAS 500 (Cytiva). Band intensities were quantified using ImageJ software, and tyrosine phosphorylation levels were normalized to the total protein levels.

### PTPRK oxidation analysis

For oxidation analysis, HCT116 cells transfected with PTPRK expression constructs were treated with 1 mM hydrogen peroxide (H₂O₂) for 15 min prior to harvest. Immunoprecipitation was performed using 2 μg of anti-FLAG M2 monoclonal antibody (F1804, Sigma-Aldrich/Merck, Germany). The immunoprecipitated proteins were separated by SDS–PAGE and subjected to Western blot analysis. Oxidation of the PTP active site was detected using an anti-oxidized protein tyrosine phosphatase (PTP) active site monoclonal antibody (R&D Systems, catalogue no. MAB2844).

Band intensities were quantified using ImageJ software (https://imagej.nih.gov/ij/), and the levels of oxidized PTP active site were normalized to total PTPRK protein levels.

### Co-immunoprecipitation analysis

HCT116 and LS411N cells were transfected with either the PTPRK construct vector or a FLAG-HA-GFP vector. After 72 hours of incubation, cells were lysed in lysis buffer (#9803, Cell Signalling Technology). The lysates were centrifuged, and 2 μg of following antibodies were added to each sample: anti-FLAG M2 monoclonal antibody (mAb) (F1804, Sigma-Aldrich/Merck, Germany), anti-ITGB4 polyclonal antibody (21738-1-AP, 1:1000, Proteintech), mouse IgG1 Isotype control (G3A1, #5415, Cell Signalling Technology) or normal rabbit IgG (#2729, Cell Signalling Technology). Samples were incubated at room temperature for 10 minutes. Next, 50 μl of Dynabeads™ Protein G (Veritas, Tokyo, Japan) were added, and the mixture was incubated. The beads were then washed three times with PBS and boiled in 1x Laemmli buffer (BioRad) at 95 °C for 5 minutes to elute the bound proteins. The eluted proteins were analysed by SDS-PAGE followed by Western blotting.

### Cell proliferation analysis

Cells were seeded at a density of 4000 per well in 96-well plates and incubated in the IncuCyte SX5 Live-Cell Analysis System (Sartorius, Göttingen, Germany), which captured real-time images every 12 hours for 48 hours. Cell confluence was assessed using IncuCyte Basic Analyzer software (Sartorius).

### Mouse xenograft model

All animal experiments were conducted in accordance with protocols approved by the Institutional Animal Care and Use Committee. Female BALB/cSlc-nu/nu mice (6 weeks old) were purchased from Japan SLC (Hamamatsu, Japan). HT29 or HT29^R1122C^ cells (1×10^6^ cells) were injected subcutaneously into the flanks of these mice. The tumour size was measured every 2–3 days, and the tumour volume was calculated using the formula: volume = (width^2^ × length)/2. On day 34 after tumour injection, mice were sacrificed, and tumours were excised and weighed.

### Immunohistochemistry analysis

Formalin-fixed, paraffine-embedded tissue sections were deparaffinized and subjected to antigen retrieval using tris/EDTA buffer, pH 9.0 (ImmunoActive IA9500; Matsunami Glass, Osaka, Japan). After blocking endogenous peroxidase activity with 3% H2O2 for 20 min, the sections were incubated with the primary antibodies for overnight at 4°C. The following primary antibodies were used: anti–phospho-ITGB4 (Tyr1510) antibody (GTX55321; 1:100 dilution; GeneTex, Irvine, CA, USA) and anti-mouse CD45 antibody (157,602; 1:100; BioLegend, San Diego, CA, USA). Subsequently, the sections were incubated with HRP-conjugated secondary antibody, followed by visualization with 3,3′-diaminobenzidine (Nichirei Bioscience, Tokyo, Japan) and counterstaining with haematoxylin. Images were acquired using a BZ-X810 microscope (KEYENCE, Osaka, Japan). DAB staining intensity was quantified using ImageJ software by measuring the mean grey value.

### Statistical analysis

Statistical analysis was performed using JMP Pro version 13.0.0 (SAS institute, Cary, NC, USA). Categorical variables were compared between tumour groups using Fisher’s exact test. All reported *P*-values were two-tailed, and statistical significance was defined as *P* < 0.05.

## Results

### Identification of *PTPRK* mutations in DCs

To identify specific gene alterations contributing DC formation, we initially performed WES analysis in seven DCs (Supplementary Table S2) and corresponding normal tissues used as a control. These samples yielded 5895 mutations in exonic regions, among which 3946 were non-synonymous mutations corresponding to 3200 genes (Fig. 1a). In sample D005, a mutation was detected in the exonuclease domain of the DNA polymerase epsilon (*POLE*) gene, which engenders hypermutation in CRCs with microsatellite stability (MSS). Consistent with this, an extremely high number of mutations were observed in this sample (Fig. 1a, Supplementary Table S2) *(**2**,*  *23**)*. Although samples D003 and D007 have non-synonymous mutations outside of the exonuclease domain in the *POLE*, the number of mutations in both cases was lower compared to D005 (Fig. 1a, Supplementary Table S2).

**Fig. 1:**
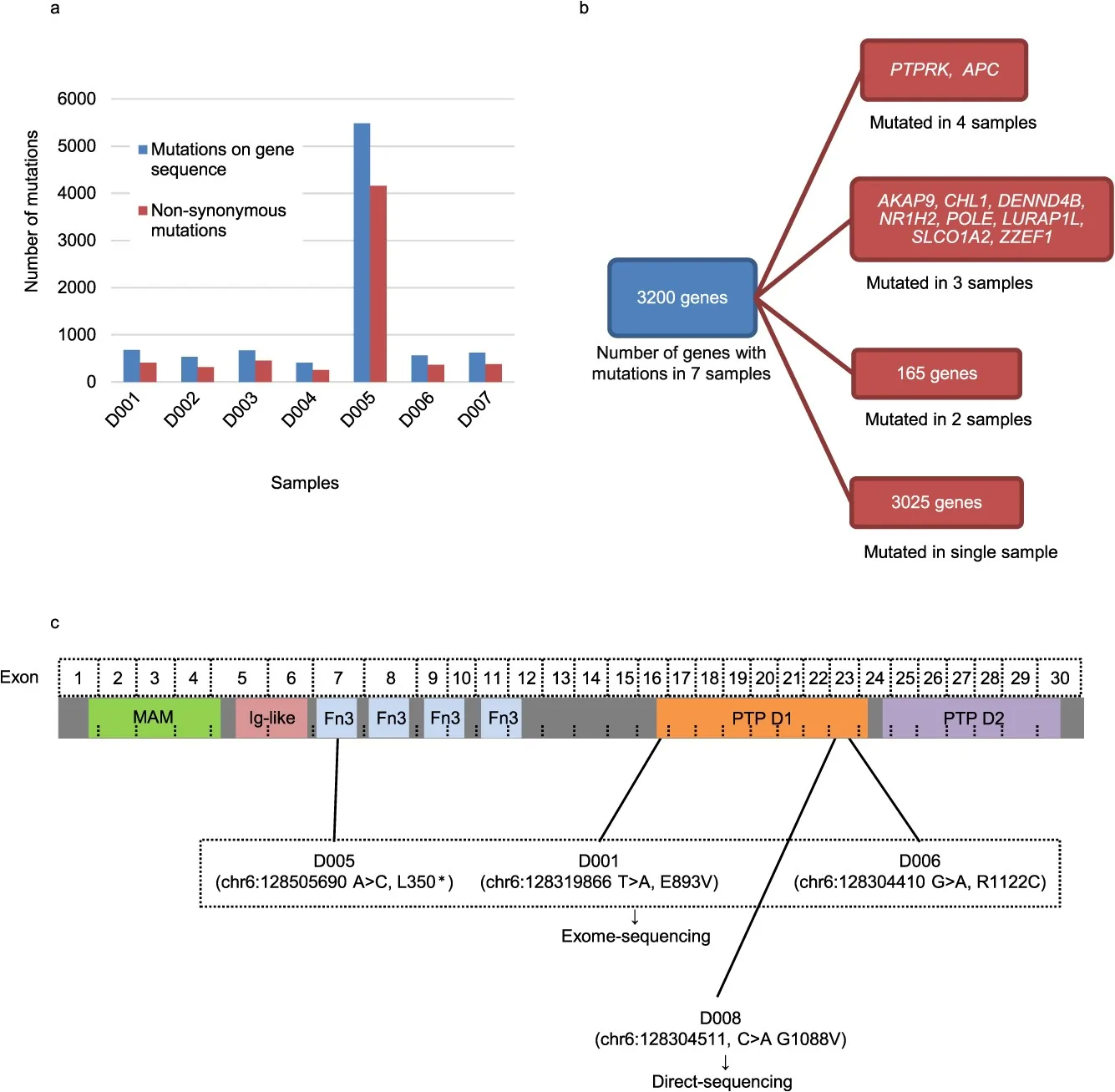
**Exome sequencing and distribution of PTPRK mutations.** (a) Number of mutations identified by whole-exome sequencing in seven DC samples. Blue and red bars indicate the total number of mutations in gene regions (5′ and 3′ untranslated regions, exons and introns) and non-synonymous mutations, respectively. (b) A total of 3200 gene mutations were identified across the seven samples. Among these, mutations in *PTPRK* and *APC* were detected in four samples. Other gene mutations were found in three, two or a single sample(s). (c) Genomic locations of *PTPRK* mutations identified through sequencing analysis. Domains of PTPRK: MAM, meprin-A5 protein-receptor protein tyrosine phosphatase μ domain; Ig-like, immunoglobulin-like domain; Fn3, fibronectin type-III domain; PTP D1, protein tyrosine phosphatase domain 1; PTP D2, protein tyrosine phosphatase domain 2. DNA alteration: chr, chromosome; A, adenine; C, cytosine; T, thymine; G, guanine. Amino acid change: L, leucine; E, glutamic acid; V, valine; G, glycine; R, arginine; C, cysteine; *, stop codon.

Notably, among the 3946 non-synonymous mutations, *APC* and *PTPRK* genes were most frequently mutated (four out of seven DCs, Fig. 1b). Three out of four *PTPRK* mutations in D001 (chr6:128319866 T > A, E893V), D005 (chr6:128505690 A > C, L350^*^) and D006 (chr6:128304410 G > A, R1122C) were cancer-specific, while a *PTPRK* mutation (chr6:128294824 G > A) was found in both normal and cancerous tissues from patient D007, indicating that this mutation may reflect an ongoing process of field cancerization *(**24**)*. These mutations were further validated with pyrosequencing analysis (Supplementary Fig. S2). Among three mutations, two were in the coding region of cytoplasmic PTP D1 domain and one was in the fibronectin type-III (Fn3) domain (Fig. 1c, Supplementary Table S2). A previous large-cohort genome-wide mutation analysis revealed that *APC* mutations were frequently observed across CRC subtypes, whereas *PTPRK* mutations were much less frequent *(**2**)*. Together with our current data, this suggests a particular importance of *PTPRK* mutations in DC tumorigenesis.

### Additional identification of PTPRK mutations in DC samples

Given our initial results, we further examined *PTPRK* exons 7 and 16–24, which correspond to the coding regions of Fn3 and D1 domain. Among 15 additional DC samples, we found one DC (D008) had a mutation (G1088V) in the D1 domain and two samples (D016, D018) had mutations in the intron separating exon 23 and 24, which encode *PTPRK* D1 domain (Fig. 1c, Supplementary Table S2, Supplementary Fig. S3). Due to a lack of matched normal tissue, we were unable to determine whether these were somatic intronic mutations. In total, four cancer-specific *PTPRK* mutations (three in the D1 domain and one in the Fn3 domain) were found among 22 DCs (18.2%, Supplementary Table S2). Interestingly, these four samples harbour mutations in other well-known CRC genes, such a, *TP53*, *KRAS*, *APC* and *POLE*, and all samples had MSS status (Supplementary Table S2).

### Clinical and molecular characteristics of PTPRK-mutant CRCs in public databases

Using public databases (TCGA and COSMIC), we next analysed the clinical characteristics of cancer patient samples with *PTPRK* mutations (Supplementary Table S3). Among 567 CRC cases, 63 *PTPRK* mutations were identified in 51 tumours (9.0%). The majority of *PTPRK* mutations were located in the D1 domain (15 mutations, 23.8%), D2 domain (11 mutations, 17.5%), or Fn3 domains (9 mutations, 14.3%) (Supplementary Fig. S4, Supplementary Table S3).

PTPRK-mutant CRCs were significantly more prevalent in the proximal colon (32/213, 15.0%) than in the distal colon (10/274, 3.6%) (*P* < 0.01, Table I, Fig. 2a,). Furthermore, PTPRK mutations were more frequently observed in CIMP-positive (CIMP-P) (11/87, 12.6%) and MSI (33/141, 23.4%) cases compare to CIMP-negative (CIMP-N) (5/134, 3.7%) and MSS (14/288, 4.9%) cases, respectively (both *P* < 0.01, Table II, Fig. 2a).

**Table I:** Clinicopathological features of CRCs with *PTPRK* mutations and *PTPRK* WT cases in the public database

	Number of cases	*PTPRK* Mut	*PTPRK* Wt	*P*-value
Gender				
Male	147 (53.8)	23 (15.6)	124 (84.4)	NS
Female	126 (46.2)	20 (15.9)	106 (84.1)	
Tumour location^*^				
Proximal	213 (43.7)	32 (15.0)	181 (85.0)	<0.0001
Distal	274 (56.3)	10 (3.6)	264 (96.4)	
CIMP status				
CIMP-P	87 (38.5)	11 (12.6)	76 (87.4)	0.0148
CIMP-N	139 (61.5)	5 (3.6)	134 (96.4)	
MSI status				
MSI	141 (32.9)	33 (23.4)	108 (76.6)	<0.0001
MSS	288 (67.1)	14 (4.9)	274 (95.1)	
CCS cluster				
CCS1	135 (57.4)	3 (2.2)	132 (97.8)	0.0001
CCS2	39 (16.6)	7 (17.9)	32 (82.1)	
CCS3	61 (26.0)	10 (16.4)	51 (83.6)	

Values in parentheses indicate the percentages. ^*^Proximal: cecum, ascending and transverse colon; Distal: descending and sigmoid colon and rectum. Mut, mutant; Wt, wild type; CIMP-P, CpG island methylator phenotype positive; CIMP-N, CpG island methylator phenotype negative; MSI, microsatellite instable; MSS, microsatellite stable; CCS, colon cancer subtype; NS, not significant.

**Fig. 2:**
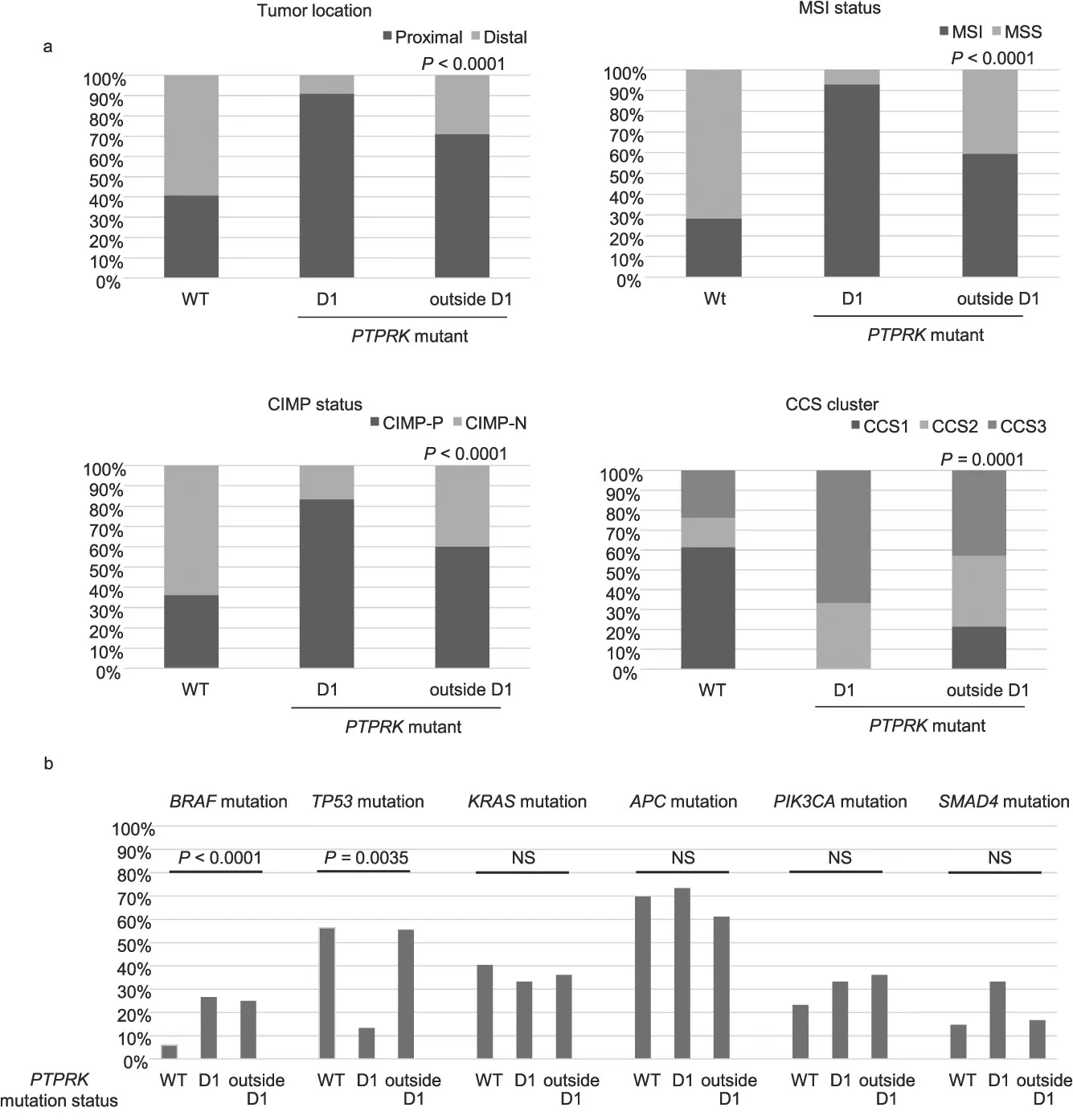
**Characteristics of *PTPRK*-mutant and** WT **CRCs analysed using public databases.** (a) Association between PTPRK mutation status and major clinicopathological features. (b) Co-occurrence patterns of PTPRK mutations with alterations in *BRAF*, *TP53*, *KRAS*, *APC*, *PIK3CA* and *SMAD4*. The *x*-axis shows PTPRK mutation categories, and the *y*-axis indicates the proportion of tumours within each category. CIMP-P, CpG island methylator phenotype positive; CIMP-N, CpG island methylator phenotype negative; MSI, microsatellite instable; MSS, microsatellite stable; CCS, colon cancer subtype; D1, *PTPRK* D1 domain mutant; Outside D1, *PTPRK* mutation outside of D1 domain mutant; WT, wild type.

**Table II:** Clinicopathological characteristics by PTPRK mutation status

		*PTPRK* mutation status			*P-*value
	Number of cases	D1	Outside D1	Wt	
Gender					
Male	147 (53.8)	5 (3.4)	18 (12.2)	124 (84.4)	NS
Female	126 (46.2)	6 (4.8)	14 (11.1)	106 (84.1)	
Tumour location^*^					
Proximal	213 (43.7)	10 (4.7)	22 (10.3)	181 (85.0)	<0.0001
Distal	274 (56.3)	1 (0.3)	9 (3.3)	264 (96.4)	
CIMP status					
CIMP-P	87 (38.5)	5 (5.7)	6 (6.9)	76 (87.4)	0.0267
CIMP-N	139 (61.5)	1 (0.7)	4 (2.9)	134 (96.4)	
MSI status					
MSI	141 (32.9)	14 (9.9)	19 (13.5)	108 (76.6)	<0.0001
MSS	288 (67.1)	1 (0.3)	13 (4.5)	274 (95.1)	
CCS cluster					
CCS1	135 (57.4)	0 (0)	3 (2.2)	132 (97.8)	0.0003
CCS2	39 (16.6)	2 (5.1)	5 (12.8)	32 (82.1)	
CCS3	61 (26.0)	4 (6.6)	6 (9.8)	51 (83.6)	
*BRAF* V600E mutation					
Positive	44 (7.8)	4 (9.1)	9 (20.5)	31 (70.5)	<0.0001
Negative	523 (92.2)	11 (2.1)	27 (5.2)	485 (92.7)	
*TP53* mutation					
Positive	314 (55.4)	2 (0.6)	20 (6.4)	292 (93.0)	0.0035
Negative	253 (44.6)	13 (5.1)	16 (6.3)	224 (88.5)	
*KRAS* mutation					
Positive	227 (40.0)	5 (2.2)	13 (5.7)	209 (92.1)	NS
Negative	340 (60.0)	10 (2.9)	23 (6.8)	307 (90.3)	
*APC* mutation					
Positive	393 (69.3)	11 (2.8)	22 (5.6)	360 (91.6)	NS
Negative	174 (30.7)	4 (2.3)	14 (8.0)	156 (89.7)	
*PIK3CA* mutation					
Positive	138 (24.3)	5 (3.6)	13 (9.4)	120 (89.6)	NS
Negative	429 (75.7)	10 (2.3)	23 (5.4)	396 (92.3)	
*SMAD4* mutation					
Positive	87 (15.3)	5 (5.7)	6 (6.9)	76 (87.4)	NS
Negative	480 (84.7)	10 (2.1)	30 (6.3)	440 (91.7)	

Values in parentheses indicate the percentages. ^*^Proximal: cecum, ascending and transverse colon; Distal: descending and sigmoid colon and rectum. D1, *PTPRK* D1 domain mutant; Out of D1, *PTPRK* out of D1 domain mutant; Wt, wild type; CIMP-P, CpG island methylator phenotype positive; CIMP-N, CpG island methylator phenotype negative; MSI, microsatellite instable; MSS, microsatellite stable; CCS, colon cancer subtype; NS, not significant.

Mutations in the D1 domain, which plays a critical role in PTPRK function *(**25**)*, were the most frequently observed PTPRK alterations in CRCs across both the public databases and our cohort. Notably, D1 domain mutations were predominantly associated with tumours located in the proximal colon (10/11, 90.9%), with a CIMP-P phenotype (5/6, 83.3%), and with MSI status (14/15, 93.3%). In contrast, these features were less frequent among CRCs harbouring mutations in other PTPRK domains (proximal location: 22/31, 71.0%; CIMP-P: 6/10, 60.0%; MSI: 15/28, 53.6%, respectively) (Fig. 2a, Table II).

Interestingly, TP53 mutations were significantly less frequent in D1 domain mutant samples (0.6% compared to 5.1%, *P* = 0.0035; Table II, Fig. 2b). There was no significant difference in the frequency of TP53 mutations in samples with PTPRK mutations outside the D1 domain (6.4% compared to 6.3%), nor in samples with WT PTPRK (93.0% compared to 88.5%). In contrast, BRAF V600E mutations were more frequent in CRCs with PTPRK mutations than in WT CRCs (4/15 [26.7%] in D1 mutants, 9/36 [25.0%] in other mutants and 31/516 [6.0%] in WT, *P* < 0.0001) (Table II, Fig. 2b).

CRCs have been classified into three colon cancer subtypes (CCSs) based on clinical outcomes *(**4**)*. CCS3, characterized by serrated adenocarcinoma features, BRAF mutations and an MSS/CIMP-positive phenotype, is associated with a poor prognosis due to high recurrence rates. Notably, PTPRK-mutant CRCs were significantly enriched in the CCS3 subtype compared to WT tumours [10/20 (50.0%) vs. 51/215 (23.7%), *P* = 0.0001; Table I, Fig. 2a]. Among PTPRK-mutant tumours, CCS3 classification was more common in D1 domain mutants [4/6, (66.7%)] compared to other domain mutations [6/14, (42.9%)] and WT CRCs [51/215 (23.7%), *P* = 0.0003] (Table II, Fig. 2a).

To further evaluate the clinical significance of PTPRK mutations, we assessed the prognostic impact of CRCs harbouring mutations in the PTPRK D1 domain compared with WT cases using TCGA data. Kaplan–Meier survival analysis demonstrated that overall survival was significantly worse in patients with PTPRK D1 domain mutations (Log-rank test, *P* = 0.0369) (Supplementary Fig. S5).

Taken together, these findings indicate that PTPRK mutations—particularly those affecting the D1 domain—are closely associated with a distinct molecular and clinical subtype of CRC.

### Impact of PTPRK mutations on the phosphorylation of ITGB4 and EGFR

The amino acid residue R1122 of PTPRK is located within a highly conserved sequence among vertebrate PTPs and is predicted to reside in a functionally important region of the D1 domain (Supplementary Table S4) *(**26**,*  *27**)*. To examine whether the R1122C mutation affects the redox sensitivity of PTPRK, we expressed 3 × FLAG-tagged WT and R1122C mutant PTPRK in HCT116 cells and assessed oxidation of the catalytic cysteine under oxidative stress conditions. Notably, oxidation of the catalytic cysteine was significantly reduced in the R1122C mutant compared with WT PTPRK (Supplementary Fig. S8) *(**28**)*.

These observations led us to hypothesize that alteration of R1122 may affect PTPRK catalytic function. Based on this rationale, we focused on the potential functional significance of the R1122C mutation in PTPRK. To experimentally assess its impact, we introduced PTPRK R1122C into CRC cell lines HCT116 and HT29, both of which harbour WT PTPRK (Supplementary Figs S5 and S6). PTPRK R1122C (chr6:128304410 G > A), was originally identified in the D006 sample (Fig. 1c). HCT116^WT^ and HCT116^R1122C^ as well as HT29^WT^ and HT29^R1122C^ cells expressed comparable levels of PTPRK protein (Fig. 3a).

**Fig. 3:**
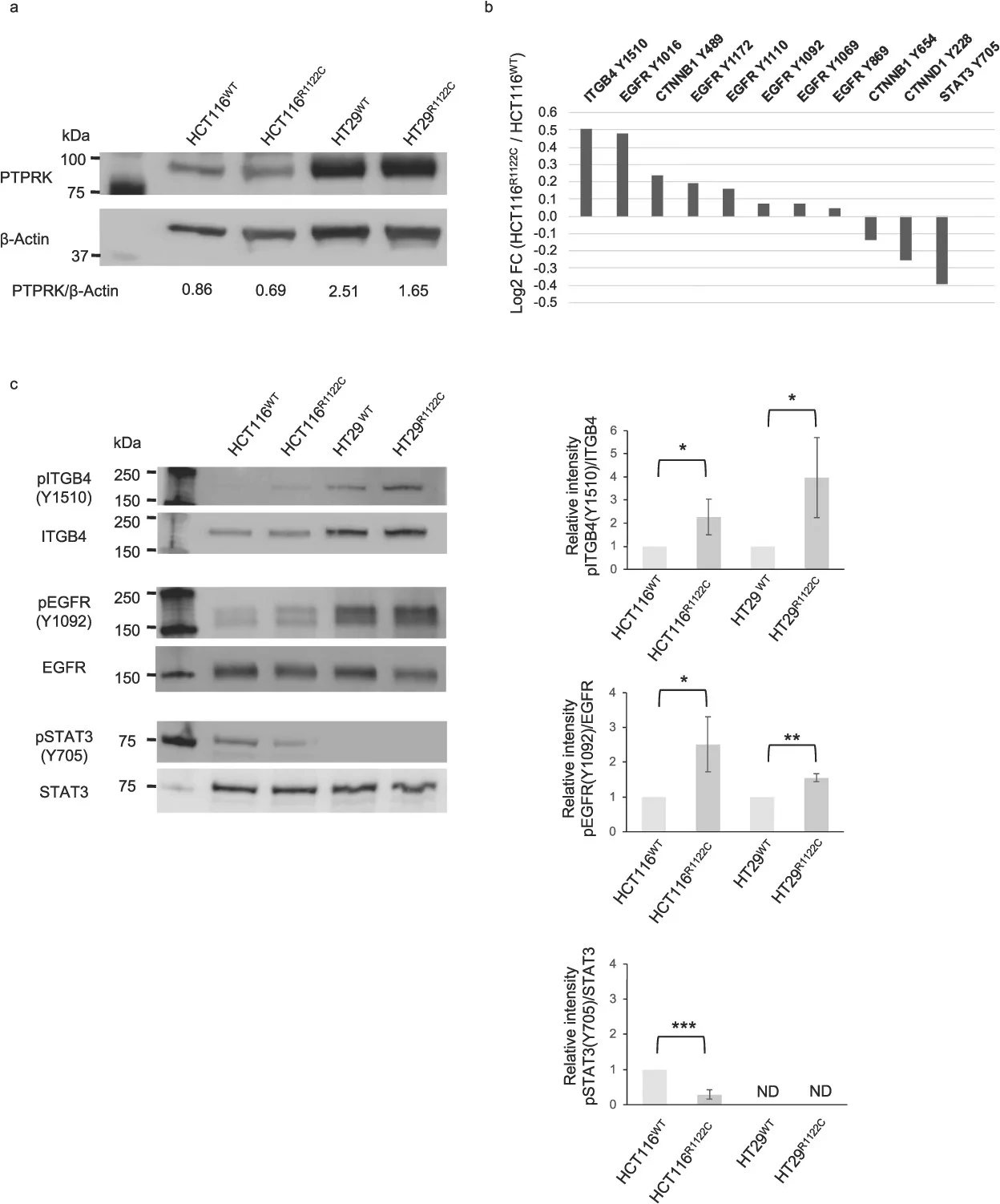
**Effect of PTPRK mutation on phosphorylation levels of target proteins.** (a) Expression levels of PTPRK in WT and mutant cells were analysed by western blotting with an anti-PTPRK antibody. β-actin was used as a loading control. Band densities of PTPRK were normalized to those of β-actin (PTPRK/β-actin), and the relative values are shown below the blot. (b) Phosphorylation levels of PTPRK substrate proteins were analysed using the Phospho Explorer Antibody Array PEX100. The *y*-axis represents the ratio of tyrosine phosphorylation levels in HCT116^WT^ to those in HCT116^R1122C^ mutant cells. (c) Left panel, phosphorylation levels of PTPRK substrates were examined in WT and R1122C mutant cells by western blotting. Specific antibodies against pITGB4-Y1510, pEGFR-Y1092 and pSTAT3-Y705 were used. Right panel, phosphorylated protein levels were normalized to the levels of their respective total proteins. Data are representative three independent experiments. Error bars indicate standard deviation (s.d.) (*n* = 3). ^*^P < 0.05, ^**^P < 0.01, ^***^P < 0.001, Student’s *t*-test. FC, fold change; ND, not detected.

We performed a phosphorylation array analysis using protein extracts from HCT116 ^WT^ and HCT116^R1122C^ cells, employing 1318 phosphorylation-specific antibodies (Supplementary Fig. S9). No proteins exhibited more than a twofold increase in tyrosine phosphorylation in HCT116^R1122C^ compared to HCT116^WT^; however, distinct differences were observed in several previously reported PTPRK substrates (Fig. 3b) *(**29–32**)*. Specifically, phosphorylation of integrin subunit beta 4 (ITGB4) at Y1510 and epidermal growth factor receptor (EGFR) at multiple sites (Y1016, Y1172, Y1110, Y1092, Y1069 and Y869) was increased in HCT116^R1122C^ cells. In contrast, phosphorylation of signal transducer and activator of transcription 3 (STAT3) at Y705, catenin beta-1 (CTNNB1) at Y654 and catenin delta-1 (CTNND1) at Y228 was decreased, while CTNNB1 phosphorylation at Y489 was modestly increased (Fig. 3b).

Western blot analysis confirmed increased phosphorylation of ITGB4 at Y1510 and EGFR at Y1092, along with reduced phosphorylation of STAT3 at Y705 in HCT116^R1122C^, consistent with the phospho-array results (Fig. 3c). Similar alterations were observed in HT29 ^R1122C^ cells, where phosphorylation of ITGB4 Y1510 and EGFR Y1092 was increased, while phosphorylation of STAT3 Y705 was undetectable (Fig. 3c). These results suggest that the PTPRK R1122C mutation differentially affects the tyrosine phosphorylation of specific residues within its substrate proteins.

### PTPRK interacts with ITGB4

Immunoprecipitation-mass spectrometry data indicate that WT PTPRK interacts with ITGB4 *(**33**)*. However, the precise mechanism by which PTPRK regulates ITGB4 remains unclear. To further investigate this, we performed co-immunoprecipitation assays in HCT116 cells using a 3 × FLAG-tagged PTPRK expression vector. A clear interaction between PTPRK and ITGB4 was detected, whereas no interaction was observed between PTPRK and EGFR (Fig. 4a). Furthermore, no ITGB4 binding was detected in cells expressing FLAG alone, confirming that the interaction between PTPRK and ITGB4 is specific (Supplementary Fig. S10a). We next examined whether the R1122C mutation affects the ability to bind ITGB4. Co-immunoprecipitation analyses in HCT116 cells expressing either FLAG–PTPRK wild type or FLAG–PTPRK R1122C demonstrated that the mutant protein retains ITGB4-binding capacity comparable to that of WT PTPRK (Fig. 4b). To validate this observation in another cell line, we repeated the experiment in LS411N cells, which endogenously express low levels of PTPRK (Supplementary Fig. S7). As in HCT116 cells, the R1122C mutant maintained its interaction with ITGB4 (Supplementary Fig. S10b). These results indicate that the R1122C mutation in PTPRK affects the phosphorylation status of ITGB4 without affecting the interaction between the two proteins. Further studies are warranted to elucidate the molecular mechanisms by which PTPRK R1122C enhances ITGB4 phosphorylation.

**Fig. 4:**
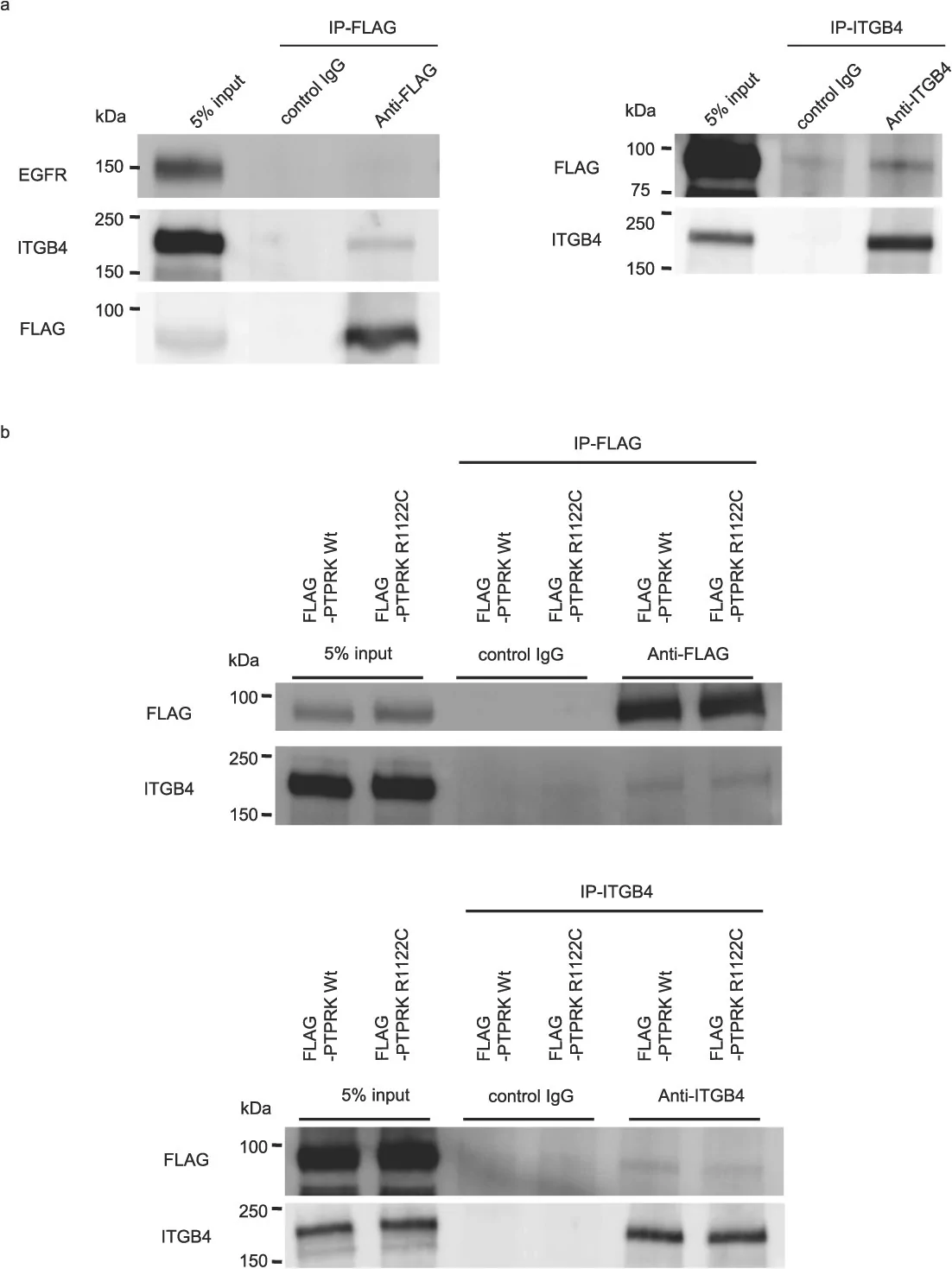
**Interaction between mutant PTPRK and ITGB4.** (a) HCT116 cells were transiently transfected with a FLAG-tagged WT *PTPRK* expression vector. After 72 h of transfection, the cells were lysed and subjected to immunoprecipitation using either anti-FLAG or anti-ITGB4 antibodies. The immunoprecipitated proteins were analysed by western blotting with indicated antibodies. (b) HCT116 cells were transiently transfected with either a FLAG-tagged WT *PTPRK* or a FLAG-tagged *PTPRK*^R1122C^ mutant expression vector. After 72 hours, the cells were lysed and subjected to immunoprecipitation using anti-FLAG or anti-ITGB4 antibodies. The immunoprecipitated proteins were then analysed by western blotting with the indicated antibodies. IP, immunoprecipitation.

### The PTPRK R1122C mutation contributes to tumour growth *In vivo*

Finally, we investigated the impact of mutant PTPRK on tumour growth. We first assessed cell proliferation *in vitro*. Under standard culture conditions R1122C had no effect on the proliferation rates of HCT116 and HT29 cell lines (Fig. 5a). We then examined the effect of the R1122C mutation on tumour growth *in vivo*. Given the more pronounced increase in ITGB4 Y1510 phosphorylation observed in HT29 cells (Fig. 3c), HT29 ^WT^ and HT29^R1122C^ cells were subcutaneously injected into nude mice (BALB/cSlc-nu/nu, *n* = 5) and tumour growth was subsequently monitored. Differences in tumour volume became evident by day 13 post-inoculation, and tumours derived from HT29^R1122C^ cells were significantly larger by day 31 (Fig. 5b). Moreover, tumours excised from the mice were significantly larger in the HT29 ^R1122C^ group compared to controls (Fig. 5c). Immunohistochemical analysis of tumour sections demonstrated that phospho-ITGB4 staining was markedly stronger in HT29^R1122C^ tumours compared with those derived from WT cells (Supplementary Fig. S11a and b). Furthermore, staining with anti-CD45 antibody revealed a significant reduction in the number of CD45-positive infiltrating immune cells at the tumour margin in the HT29^R1122C^ tumours (Supplementary Fig. S11c and d). These results indicate that the PTPRK R1122C mutation enhances tumour growth *in vivo*, possibly by interacting with (or being modulated by) factors within the tumour microenvironment.

**Fig. 5:**
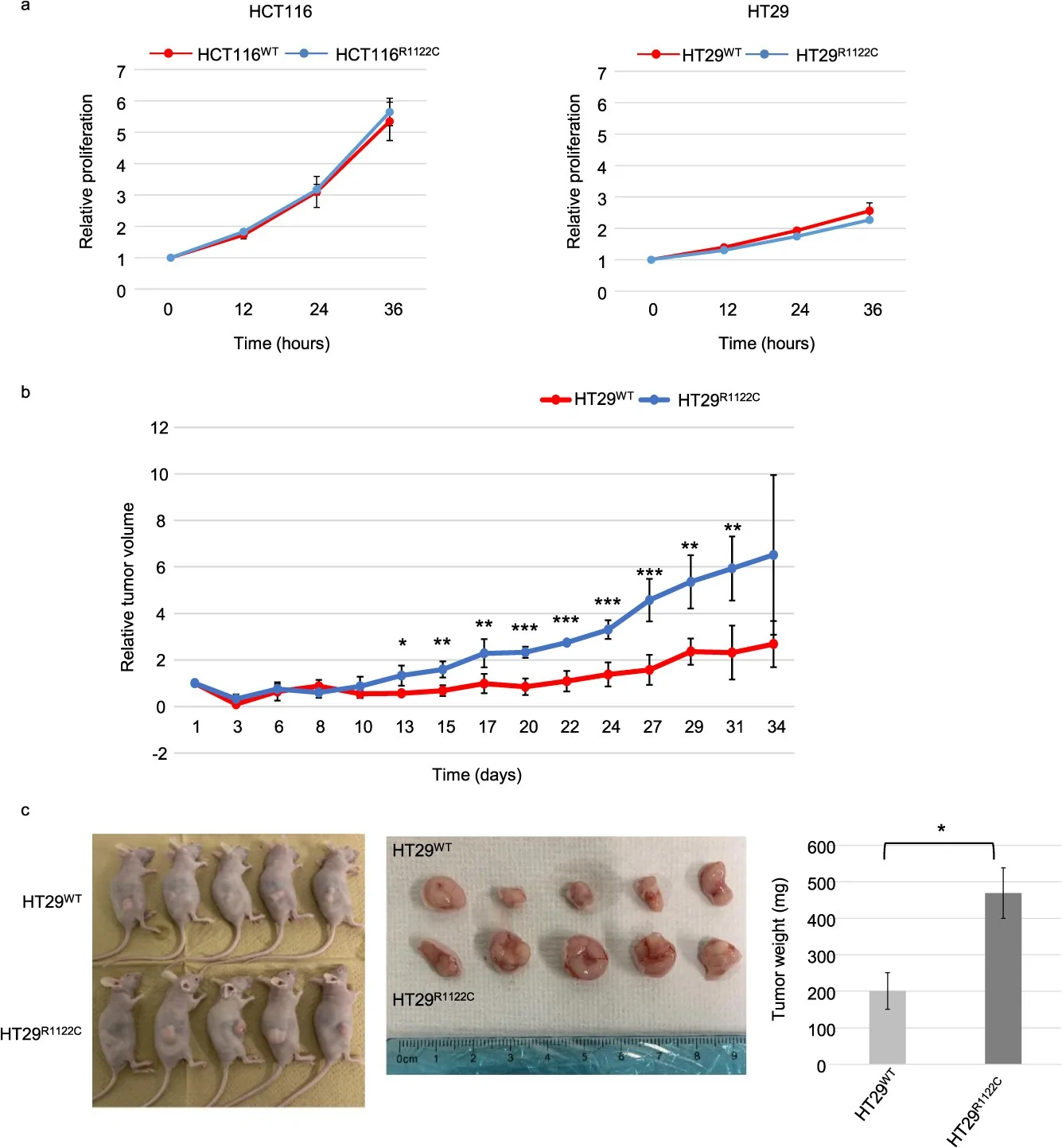
**The PTPRK R1122C mutant accelerates tumour growth *in vivo*.** (a) Proliferation rates of HCT116 and HT29 cells expressing PTPRK WT or the R1122C-mutant were analysed *in vitro*. Error bars represent standard deviation (s.d.) (*n* = 3). (b) HT29 and HT29^R1122C^ cells were subcutaneously transplanted into BALB/cSlc-nu/nu nude mice. Tumour volume was measured at each timepoint using the formula: (width^2^ × length)/2. Error bars indicate the standard deviation. (c) Tumours were harvested on day 34. The average tumour weight is shown in the right panel. The *y*-axis represents tumour weight. Error bars indicate s.d. (*n* = 5). ^*^P < 0.05, Student’s *t*-test.

## Discussion

In this study, through sequencing analysis of CRCs exhibiting a DC phenotype, we identified recurrent mutations within the D1 phosphatase domain of PTPRK, which were preferentially detected in proximal colon tumours. Furthermore, analysis of public datasets revealed that these PTPRK mutations were significantly associated with a distinctive clinical background characterized by the CpG island methylator phenotype-positive (CIMP-P), MSI and the BRAF V600E mutation. As previously reported, proximal colon cancers frequently harbour CIMP-P, MSI and BRAF V600E mutations *(**2**,*  *6**)*, and the accumulation of these alterations is implicated in colorectal tumorigenesis through the serrated neoplasia pathway *(**34**,*  *35**)*.

Notably, mutations within the D1 domain of PTPRK were mutually exclusive with TP53 mutations, one of the most common genetic alterations in CRC. Collectively, these findings suggest that PTPRK mutations may contribute to the development and progression of proximal colon cancer, predominantly through the serrated neoplasia pathway, giving rise particularly to cancers of the DC subtype.

Patients with proximal colon cancer generally have a poorer prognosis than those with distal colon cancer. Although the BRAF V600E mutation is a contributing factor, it alone does not fully account for the unfavourable outcomes observed in these patients *(**36**)*. In this study, based on the established molecular subtypes of CRC *(**3**)*, we classified our samples and found a significant association between the PTPRK mutation status and the colorectal cancer subtype (CCS). PTPRK-mutant CRCs were predominantly categorized into the CCS3-serrated and CCS2-MSI subgroups, both of which are characterized by high recurrence rates and poor prognosis. In particular, mutations within the D1 domain of PTPRK were frequently observed in the CCS3 subgroup. Consistent with our functional analyses, survival analysis of TCGA CRC cohorts demonstrated that mutations in the PTPRK D1 domain were associated with poorer overall survival. These findings suggest that PTPRK mutations, particularly those affecting the D1 domain, may be associated with poor clinical outcomes in a subset of proximal CRCs.

The PTPRK R1122C mutation identified in this study is in the D1 region of the protein, which is an essential regulator of PTPRK phosphatase activity *(**26**)*. Indeed, structural analyses have revealed that the D1 domain harbours essential catalytic motifs and highly conserved amino acid residues required for substrate dephosphorylation *(**25**,*  *27**)* (Supplementary Table S4). Although R1122 does not reside within the canonical PTP signature motif (VHCSXGXGR[T/S]G), previous biochemical and structural studies have demonstrated that conserved arginine residues surrounding the catalytic centre critically contribute to phosphatase activity by shaping the electrostatic properties of the active site *(**27**)*. Because the thiolate form of the catalytic cysteine is highly susceptible to oxidation by reactive oxygen species (ROS), including hydrogen peroxide. Thus, redox-dependent ON–OFF regulation of PTP activity is considered an important physiological mechanism *(**28**)*. In our experiments, the R1122C mutant showed markedly reduced oxidation of the catalytic cysteine compared with WT PTPRK. This finding suggests that the R1122C mutation alters the electrostatic and/or structural environment near the catalytic centre, thereby modulating the pKa and redox reactivity of the catalytic cysteine. As a result, this mutation may contribute to a constitutively reduced phosphatase activity of PTPRK. Given that WT PTPRK has tumour suppressor roles *(**37**,*  *38**)*, the clustering of mutations in the D1 domain of PTPRK in DCs, together with increased phosphorylation of ITGB4, strongly suggests that these are loss-of-function mutations that likely contribute to CRC progression.

Our phospho-antibody array analysis revealed increased phosphorylation of ITGB4 and EGFR in PTPRK R1122C mutant CRC cell lines. Although PTPRK and EGFR have been reported to interact *(**30**,*  *39**)*, we failed to reproduce a direct interaction in our CRC models, suggesting that EGFR is indirectly dephosphorylated in response to overexpression of PTPRK R1122C. In contrast, ITGB4 directly interacts with PTPRK and is dephosphorylated by it in CRC cells. These findings imply that the R1122C mutation of PTPRK may regulate ITGB4 function. Notably, ITGB4 plays a critical role in cell adhesion, migration and intracellular signalling and has been implicated as a key regulator of tumour cell migration and invasion. One of its major downstream pathways is the MEK/ERK signalling cascade, which enhances tumour cell motility and invasiveness *(**40**)*. Phosphorylation of ITGB4 at Y1510 has been shown to activate MEK1–ERK1/2 signalling and promote migration and invasion in pancreatic cancer *(**41**)*, and ITGB4-mediated MEK/ERK signalling has been implicated in modulating the efficacy of PD-1 blockade therapy in bladder cancer *(**42**)*.

In our study, the PTPRK R1122C mutation was associated with increased ITGB4 phosphorylation, implicating dysregulated ITGB4 signalling in the enhanced invasive phenotype of PTPRK-mutant DCs. In contrast, for the other identified D1-domain mutations, E893V and G1088V, there is currently limited experimental or structural evidence suggesting a direct impact on catalytic activity, although the localization of G1088V within the conserved PTP signature motif suggests potential functional relevance. Given the strong evolutionary conservation of R1122 and its established role in maintaining the catalytic environment of the active site, we therefore prioritized functional analysis of the R1122C mutation *(**43**)*.

Given that DCs frequently exhibit early invasive potential and active signalling remodelling, ITGB4 hyperphosphorylation may represent a mechanistic link between PTPRK dysfunction and disease progression. Consistent with this model, high ITGB4 expression in CRC has been associated with poorer clinical outcomes *(**44**)*.

In conclusion, we identified frequent PTPRK mutations in DC and proximal colon cancers and further elucidated their impact on downstream PTRPK substrates. We suggest that targeting ITGB4 may represent a promising therapeutic strategy for proximal CRCs harbouring PTPRK mutations.

## Supplementary Material

Web_Material_mvag024
