# Supplementary material for: Recurrent mutations in protein tyrosine phosphatase receptor-type kappa in depressed-type colorectal carcinomas
Source: J Biochem. 2026 Mar 12;180(1):49–63. doi: 10.1093/jb/mvag024 (PMC13633085; doi:10.1093/jb/mvag024)
Supplement: Supplementary file 1 [file web_material_mvag024.pdf]

Supplementary Items

1. Supplementary Figures S1 to S11

2. Supplementary Tables S1 to S4

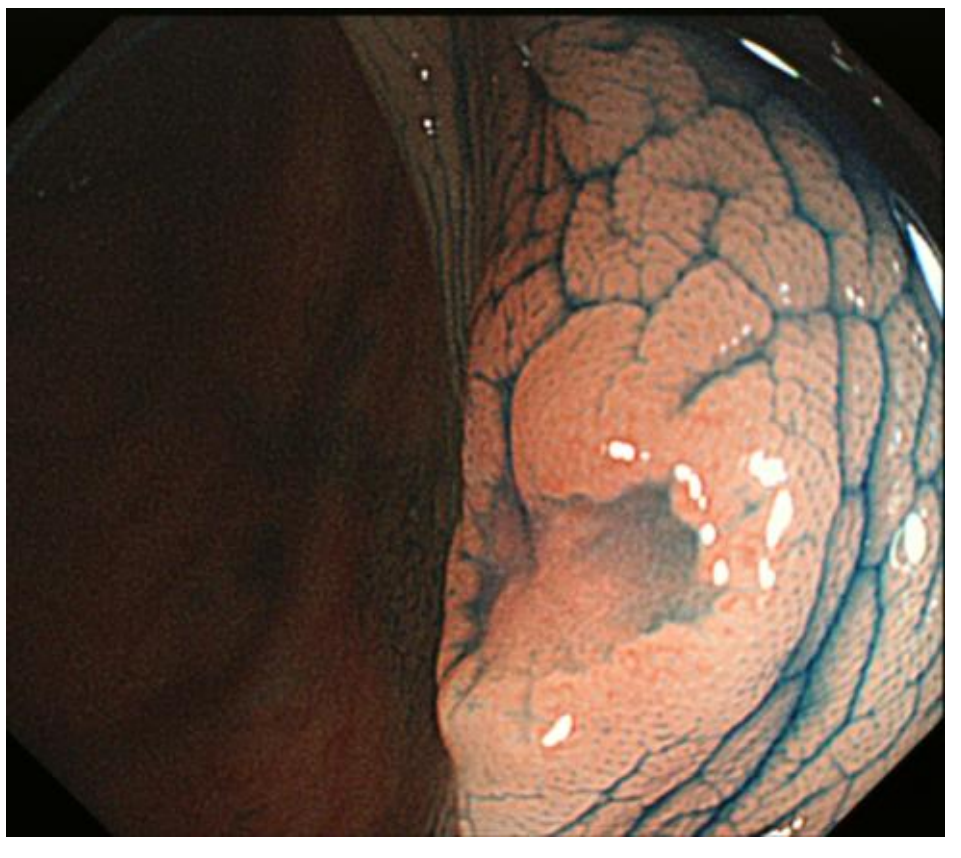

Depressed

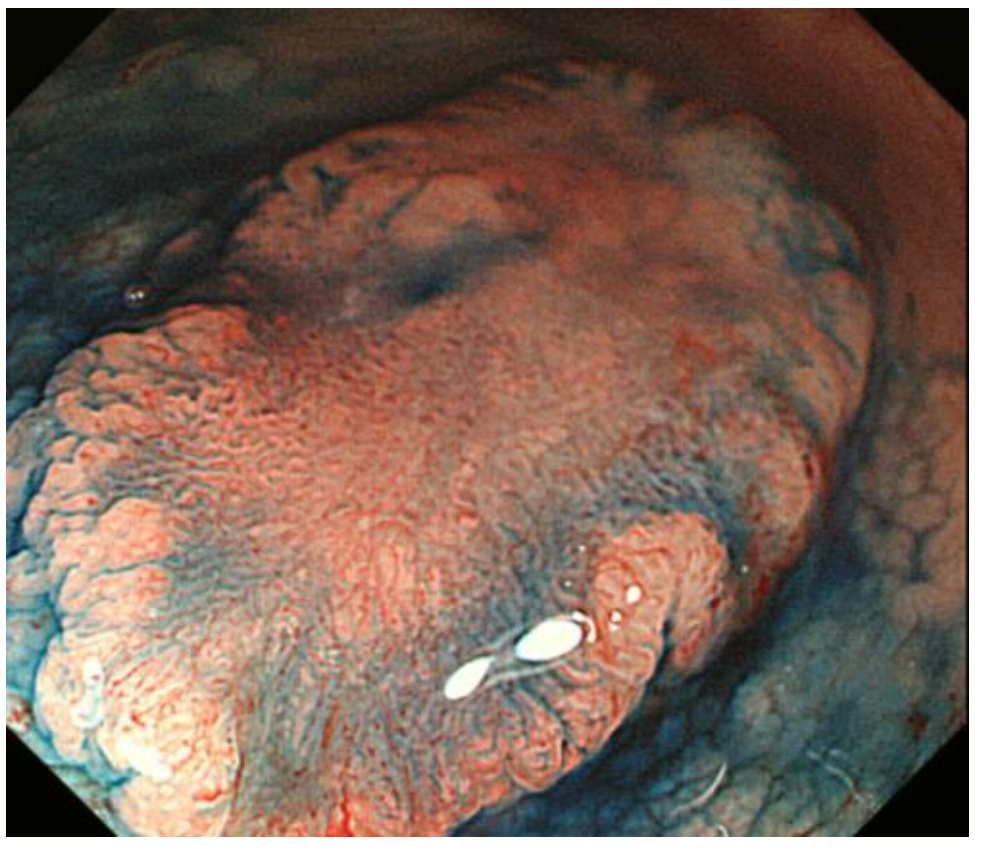

Depressed lesion with flat component

Supplementary Figure S1. Morphology of Depressed-Type Tumors
Endoscopic images of depressed-type colorectal cancers. Left: Depressed type.
Right: Depressed type with a flat component.

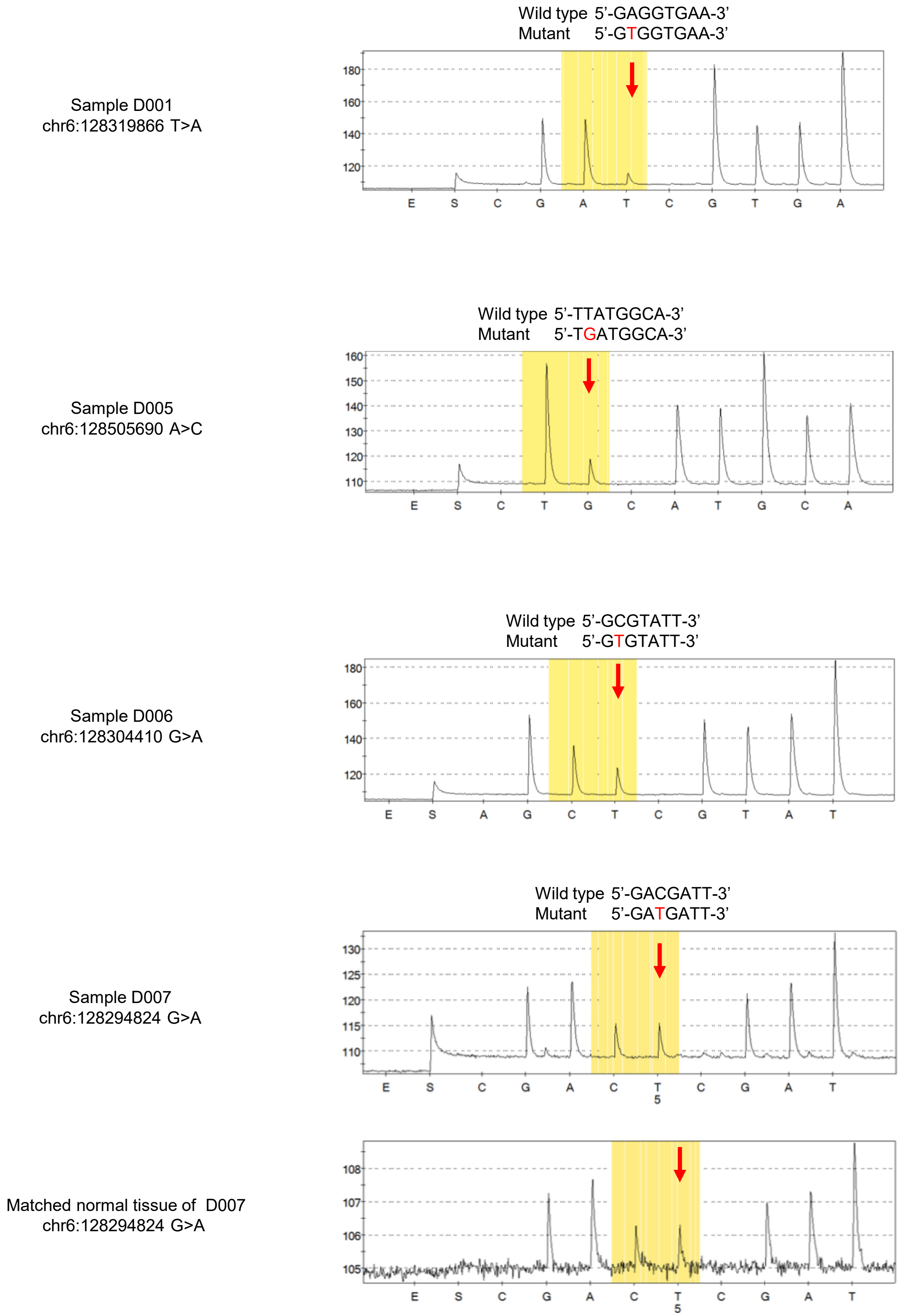

Supplementary Figure S2. *PTPRK* mutations analyzed by pyrosequencing

PTPRK mutations were analyzed using a pyrosequencer. The yellow-highlighted region indicates the mutation site.

chr, chromosome; T, thymine; A, adenine; C, cytosine; G, guanine

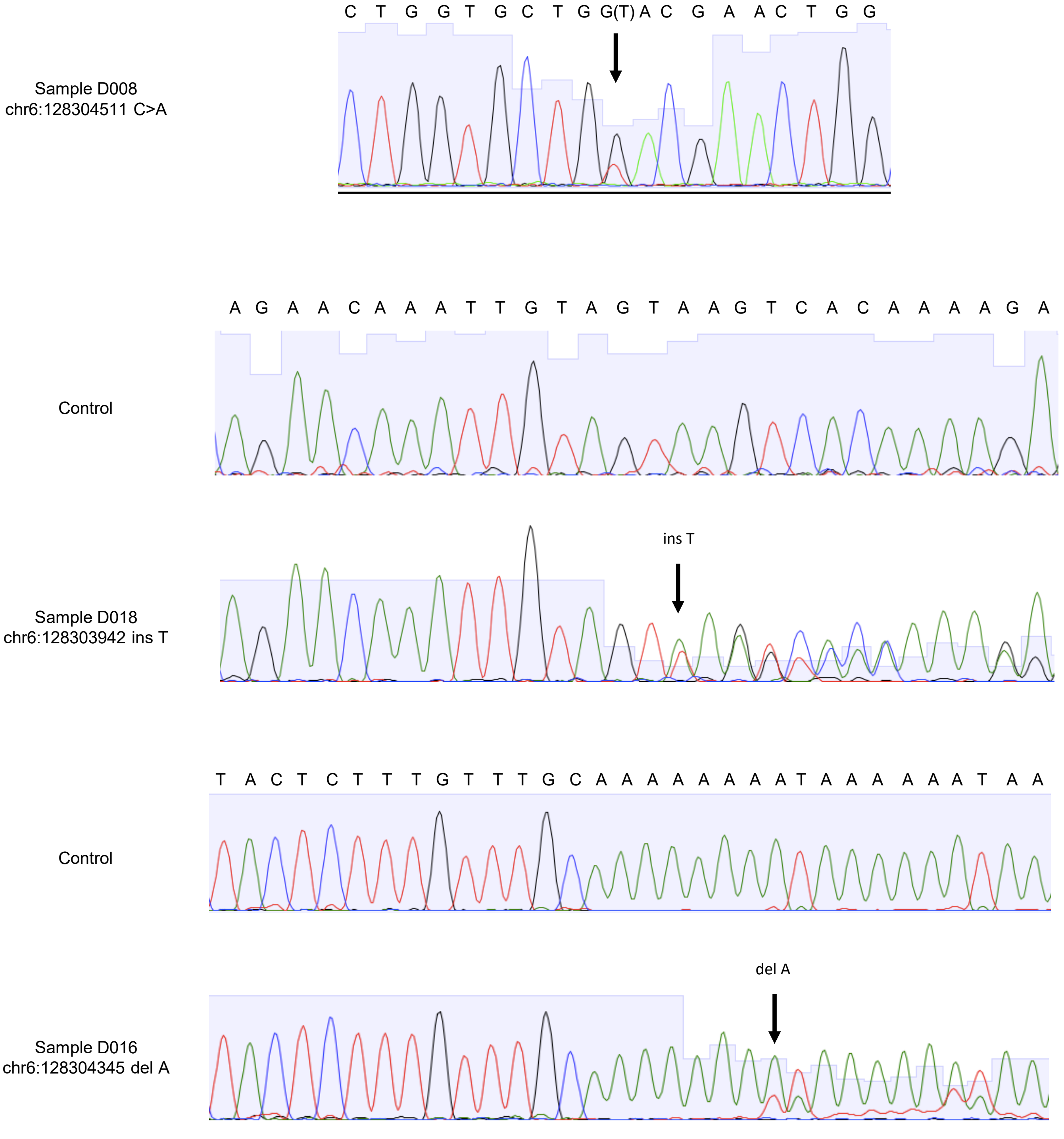

Supplementary Figure S3. *PTPRK* mutations analyzed by Sanger sequence
PTPRK mutations were analyzed using direct Sanger sequencing.
Ins, insertion; del, deletion; chr, chromosome; T, thymine; A, adenine; C, cytosine; G, guanine. Arrows indicate mutation sites.

a

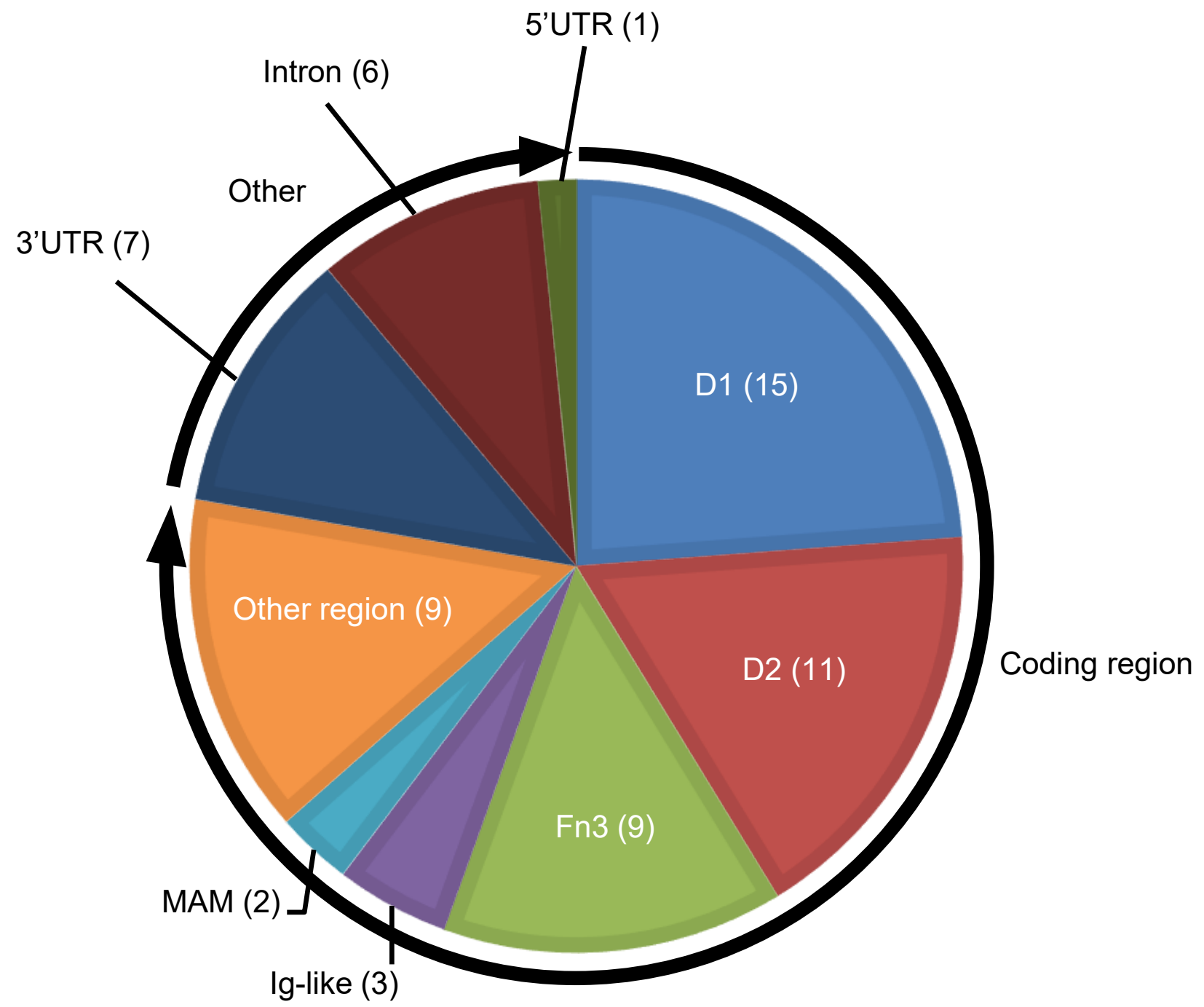

b

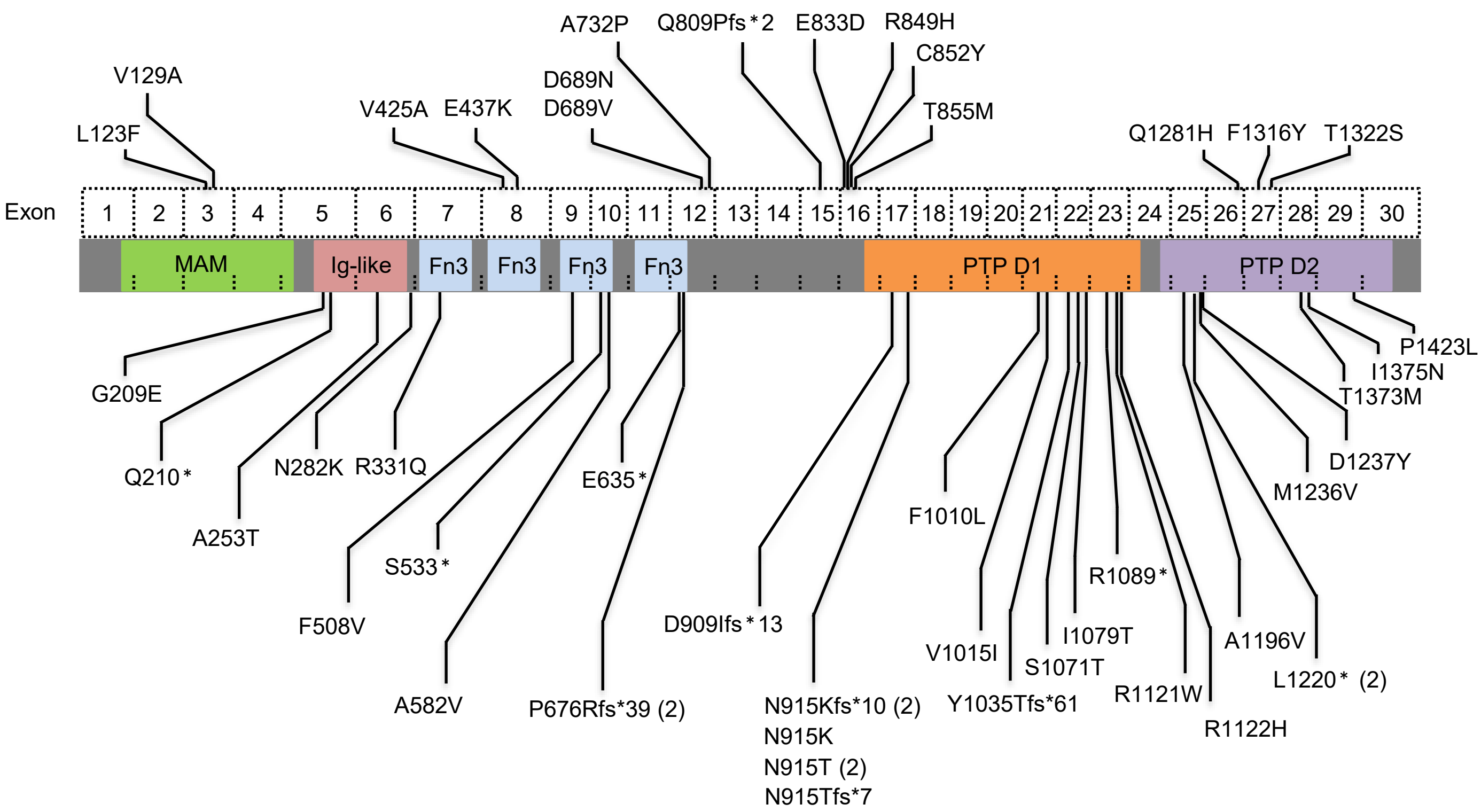

Supplementary Figure S4. *PTPRK* mutation analysis in public database

a. Pie chart showing the number of mutated samples at each location in dataset from public database (The Cancer Genome Atlas and the Catalogue of Somatic Mutations in Cancer database). MAM, meprin-A5 protein-receptor protein tyrosine phosphatase μ domain; Ig-like, immunoglobulin-like domain; Fn3, fibronectin type-III domain; PTP D1, protein tyrosine phosphatase D1 domain; PTP D2, protein tyrosine phosphatase D2 domain; 5’UTR, five prime untranslated region; 3’UTR, three prime untranslated region.

b. Schematic representation of the PTPRK protein structure and the distribution of mutations. V, valine; I, isoleuicine; P, proline; R, arginine; T, threonine; M, methionine; L, leucine; F, phenylalanine; S, serine; A, alanine; D, asparatic acid; Y, tyrosine; N, asparagine; K, lysine; W, tryptophan; Q, glutamine; G, glycine; E, glutamic acid; H, histidine; fs＊, frame shift; ＊, stop codon.

Supplementary Fig. S5

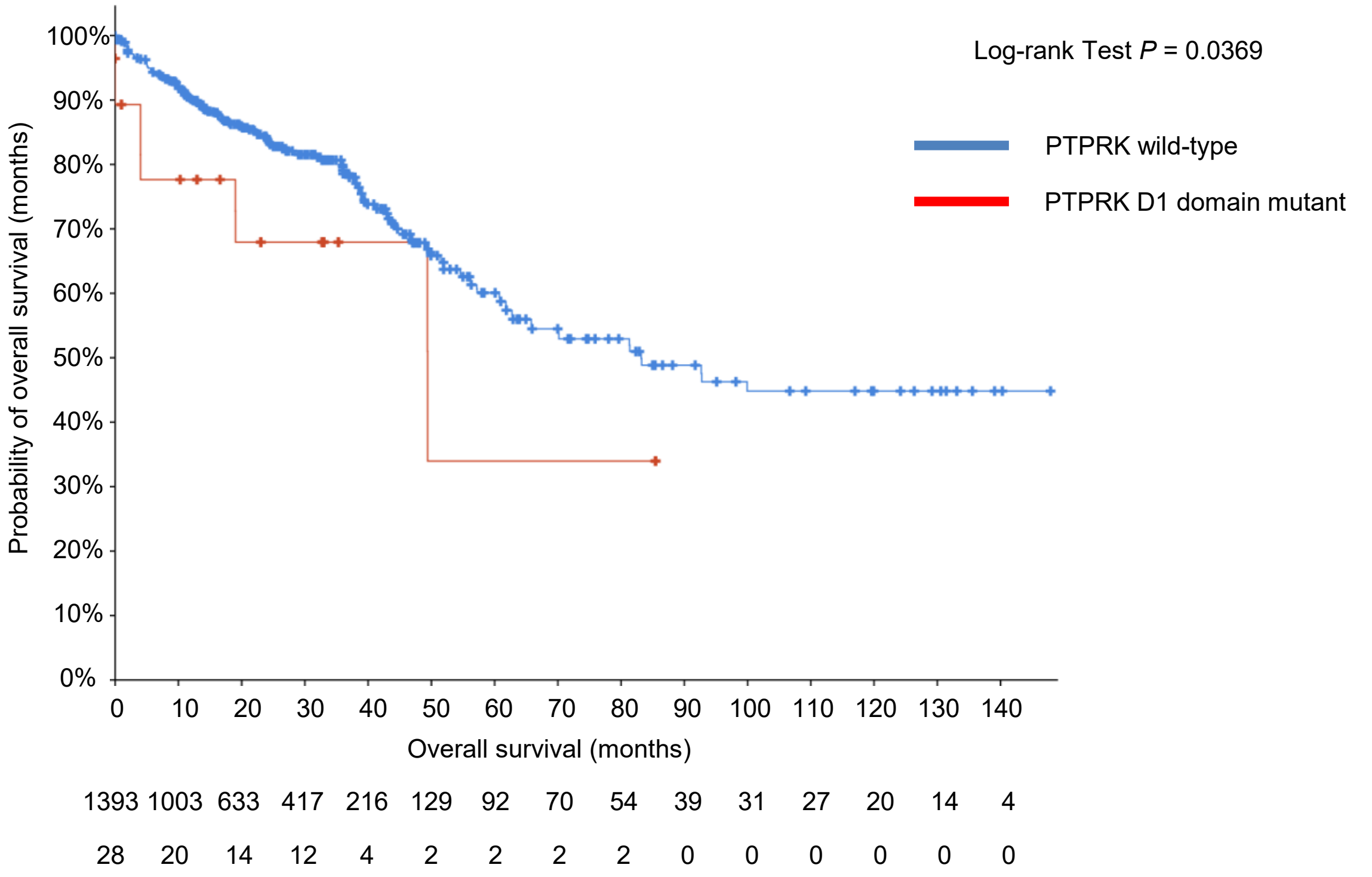

Supplementary Figure S5. Impact of PTPRK mutations on patient survival in the TCGA dataset. Kaplan–Meier survival analysis of overall survival in colorectal cancer patients from TCGA. Patients were divided into those harboring mutations within the PTPRK D1 domain and those with wild-type PTPRK. Statistical significance was assessed using the log-rank test.

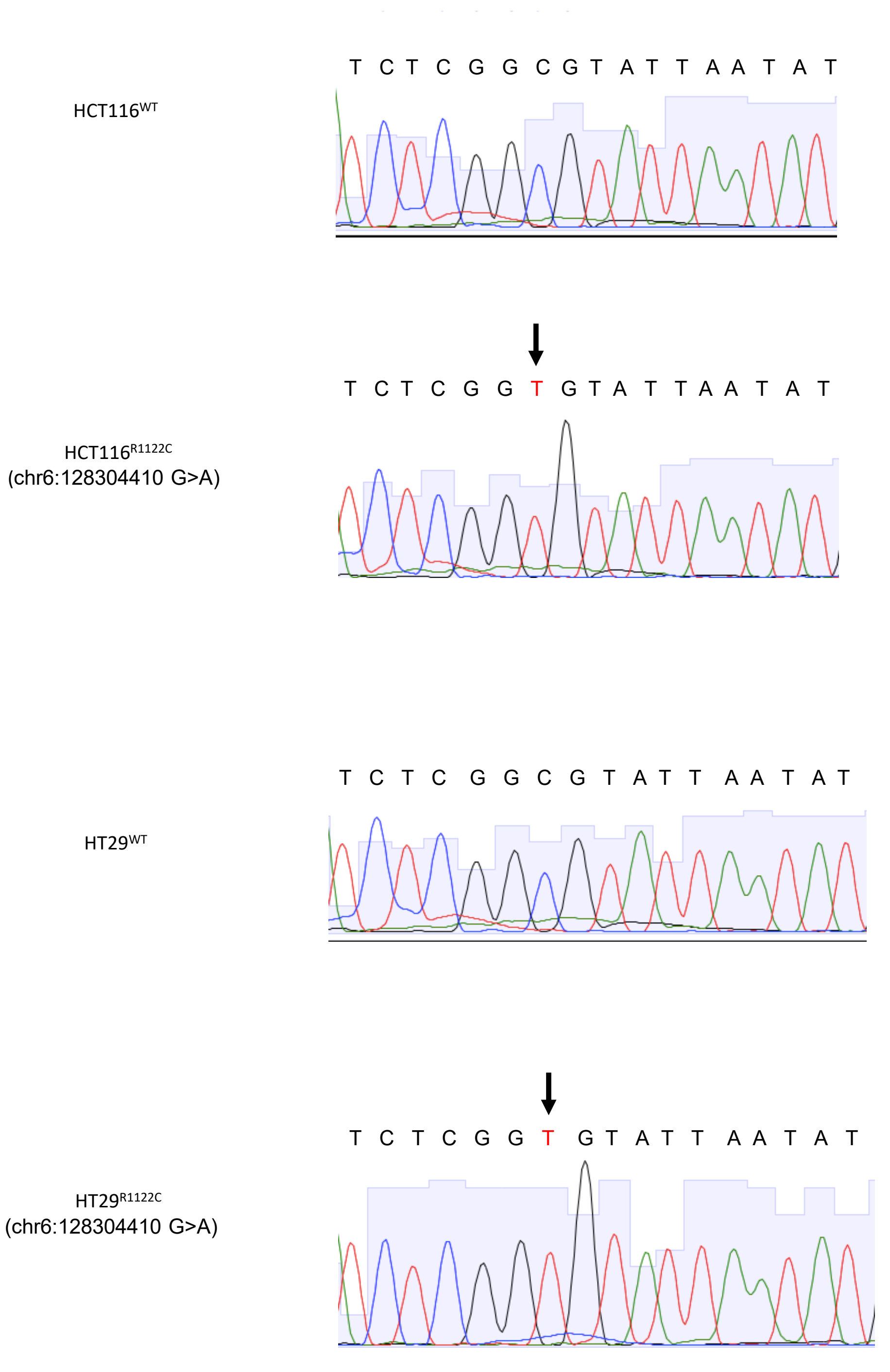

Supplementary Figure S6. PTPRK mutation was introduced by CRISPR/Cas9
PTPRK R1122 mutation was introduced using CRISPR/Cas9 system. Sanger sequencing confirmed the insertion of the mutation following genome editing. The arrows indicate the mutation site.
chr, chromosome; T, thymine; A, adenine; C, cytosine; G, guanine.

a

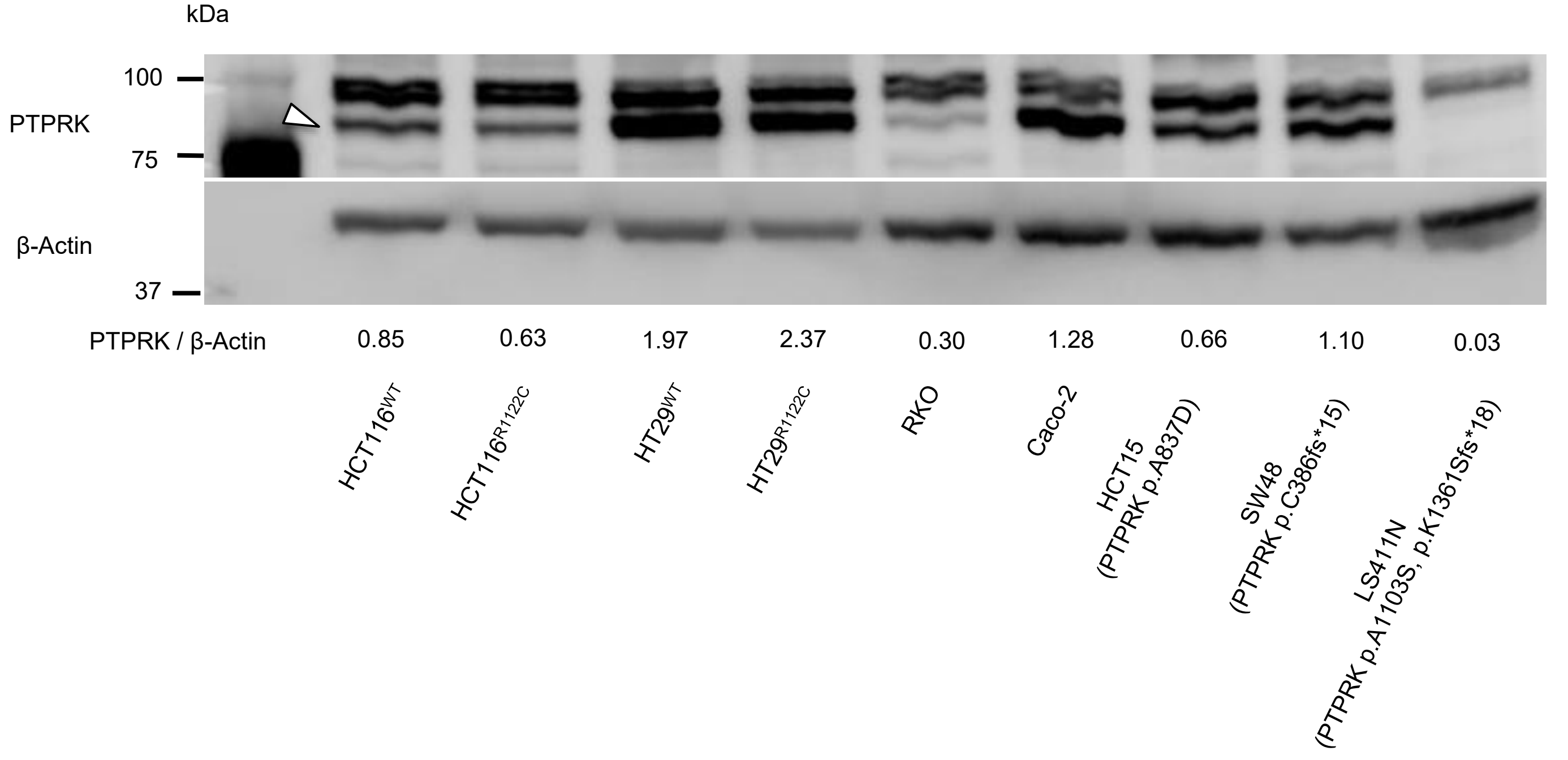

b

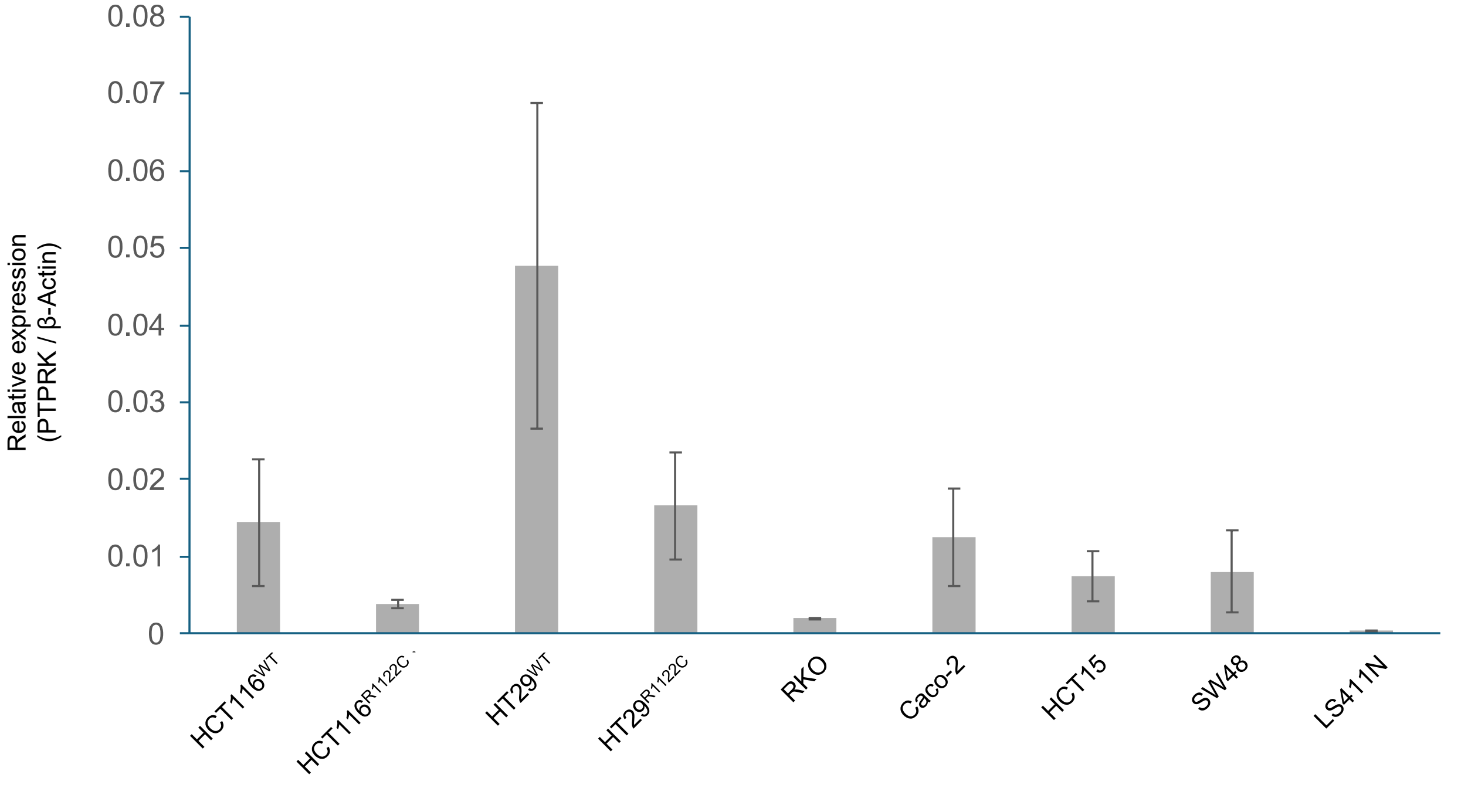

Supplementary Figure S7. PTPRK expression in colorectal cancer cell lines
a. Protein expression levels of PTPRK in colorectal cancer cell lines were analyzed by Western blotting using an anti-PTPRK antibody. β-actin was used as a loading control. Band intensities of PTPRK were normalized to those of β-actin (PTPRK/β-actin), and the relative values are shown below the blot. HCT15, SW48, and LS411N harbor PTPRK mutations as indicated. b. mRNA expression levels of PTPRK were analyzed by qPCR. β-actin was used as an internal control. The Y-axis represents expression levels relative to β-actin. Error bars indicate standard deviation (s.d.) (n = 3).

Supplementary Figure S8.

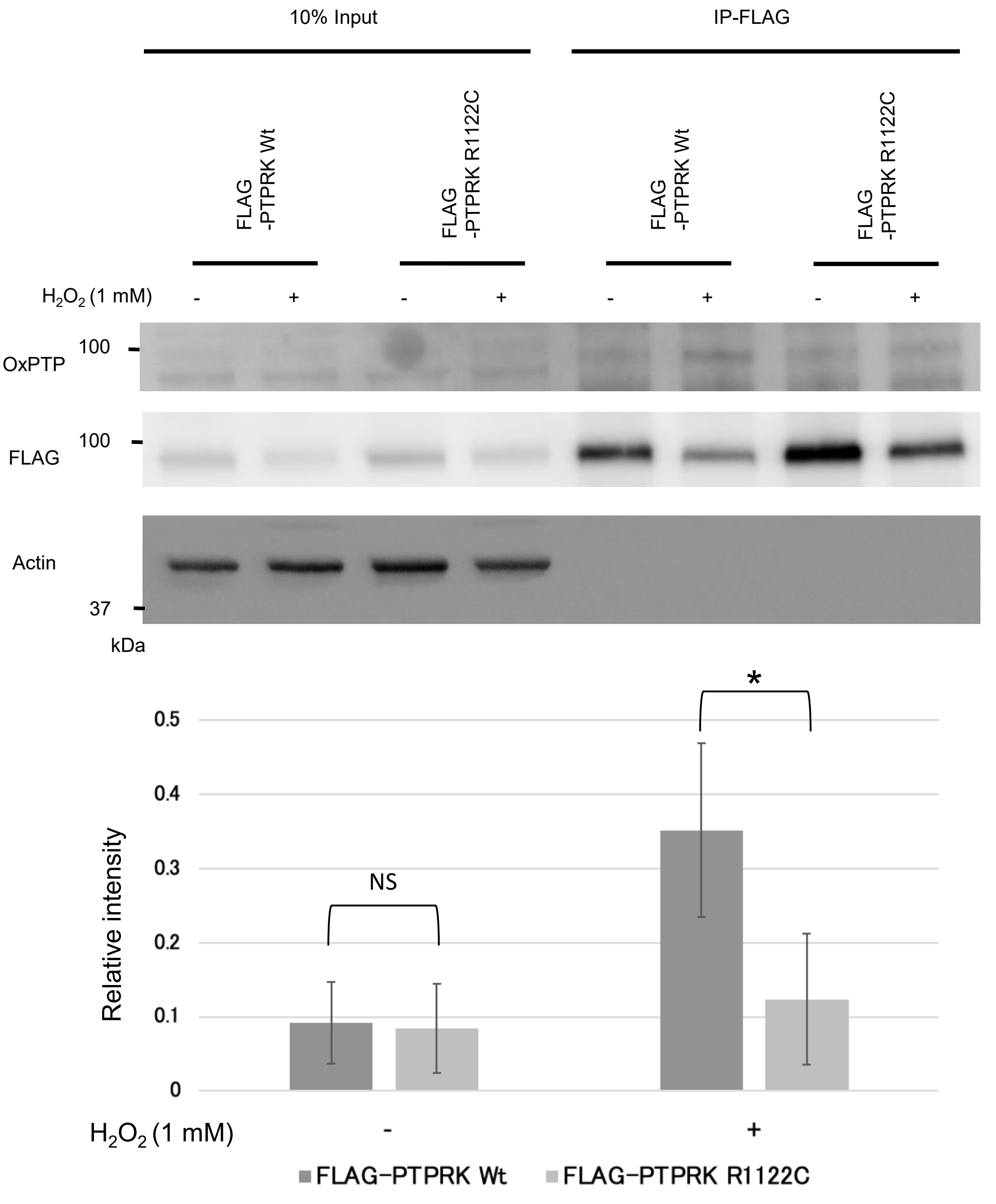

Supplementary Figure S8. Co-Immunoprecipitation analysis
HCT116 cells were transiently transfected with either a FLAG-tagged wild-type *PTPRK* or a FLAG-tagged *PTPRK*^{R1122C} mutant expression vector. After 72 hours, the cells were stimulated with or without 1 mM H_2O_2 for 15 min, lysed, and subjected to immunoprecipitation using an anti-FLAG antibody. The immunoprecipitated proteins were then analyzed by western blotting with the indicated antibodies (Upper panel). Lower panel, signal intensity of oxidized protein. Oxidized protein levels were normalized to the levels of their respective total proteins. Data are representative three independent experiments. Error bars indicate standard deviation (s.d.) (n = 3). *P < 0.01, Student's t-test.
OxPTP, oxidized protein tyrosine phosphatase; NS, not significant.

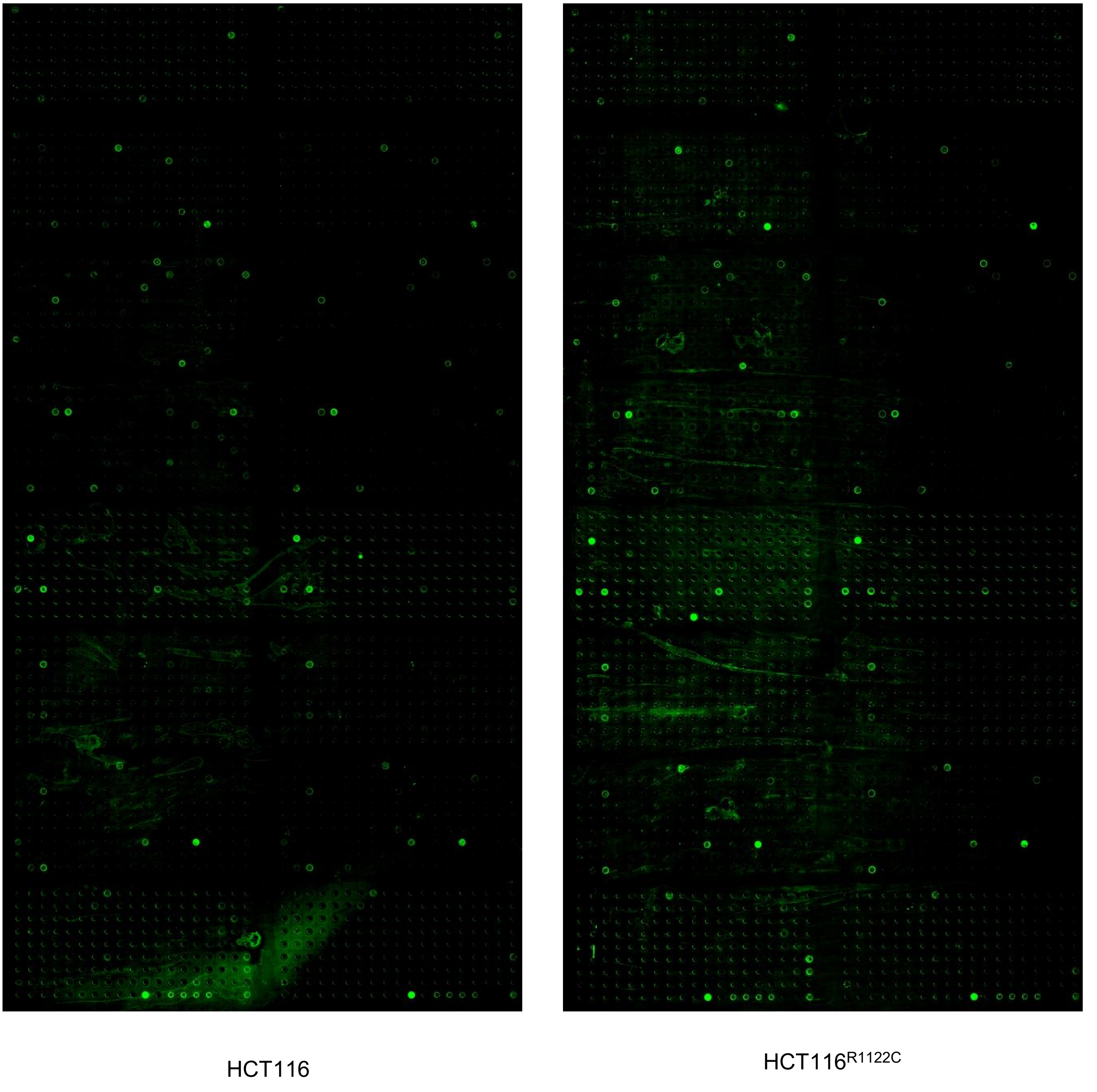

Supplementary Figure S9. Phosphorylation antibody microarray analysis
Phosphorylation profiles of HCT116 and HCT116[R1122C] cells were analyzed using the Phospho Explorer Antibody Microarray (PEX100).

Supplementary Figure S10.

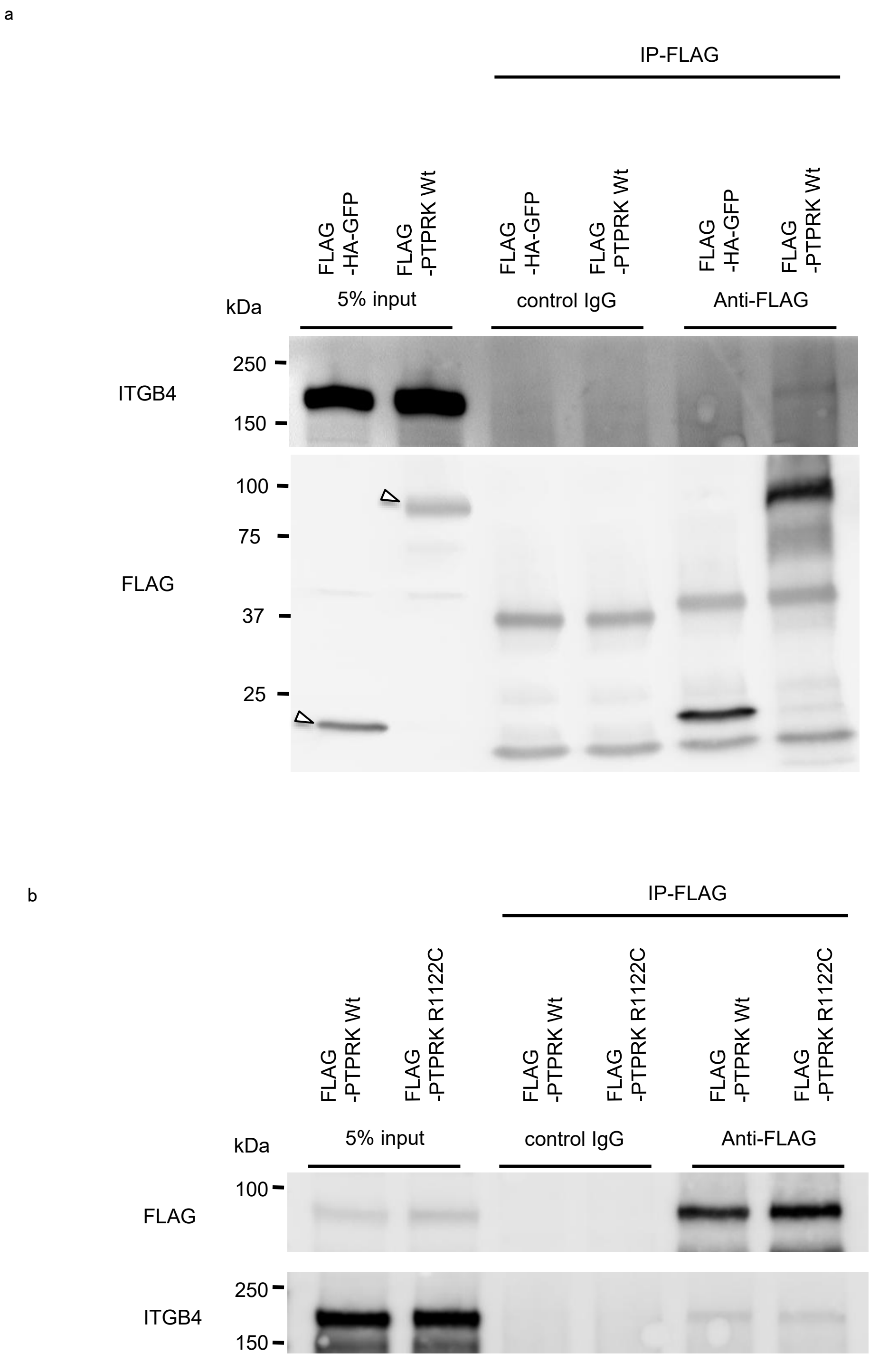

Supplementary Figure S10. Co-Immunoprecipitation analysis
a. HCT116 cells were transiently transfected with either a FLAG-tagged wild-type *PTPRK* or a FLAG-HA-GFP vector. After 72 hours, the cells were lysed and subjected to immunoprecipitation using anti-FLAG antibody. The immunoprecipitated proteins were then analyzed by western blotting with the indicated antibodies.
b. LS411N cells were transiently transfected with either a FLAG-tagged wild-type *PTPRK* or a FLAG-tagged *PTPRK*^{R1122C} mutant expression vector. After 72 hours, the cells were lysed and subjected to immunoprecipitation using anti-FLAG antibody. The immunoprecipitated proteins were then analyzed by western blotting with the indicated antibodies.

Supplementary Figure S11.

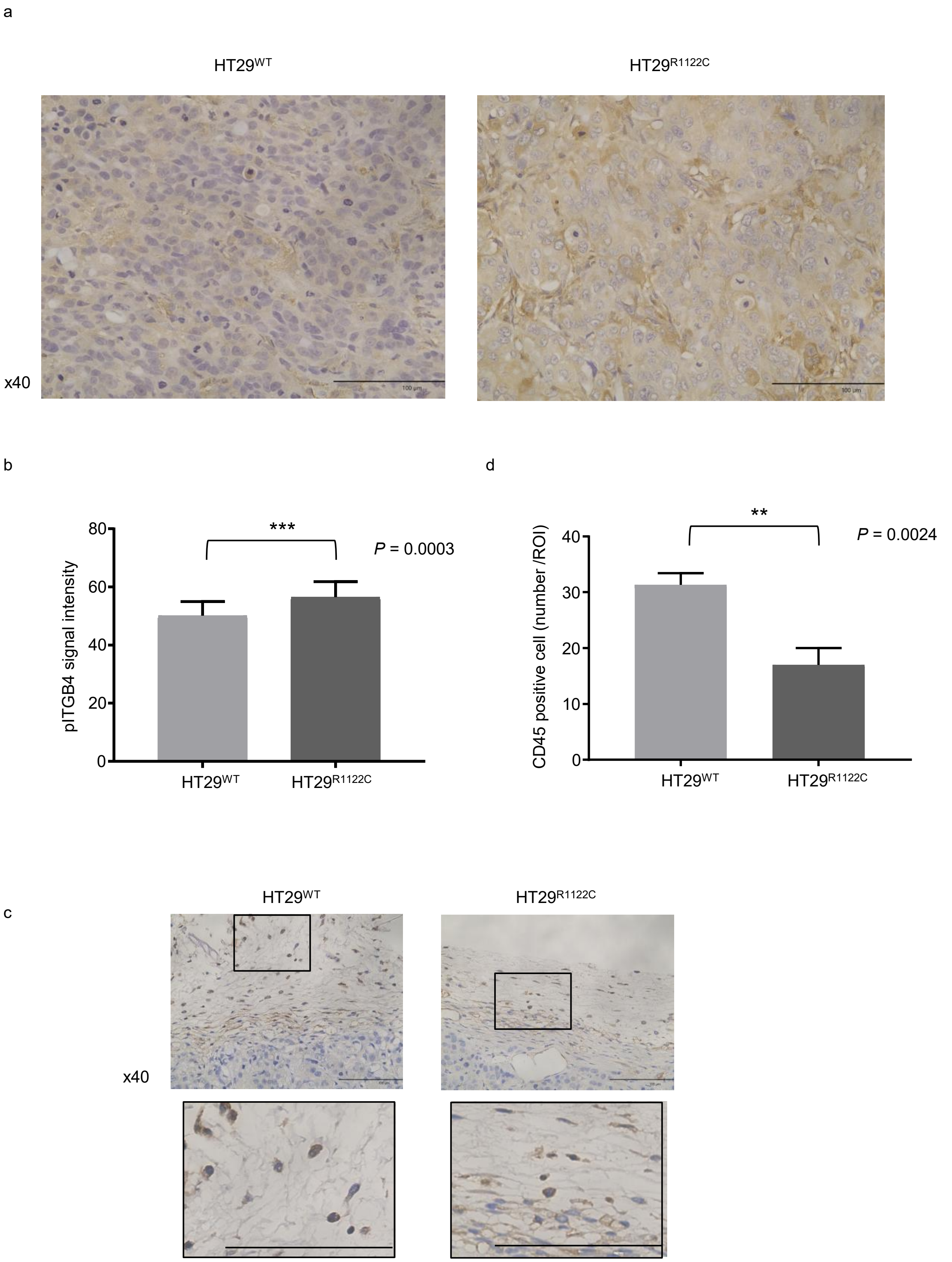

Supplementary Figure S11. Immunohistochemistry (IHC) analysis of tumors from a mouse model
a. Tumor tissues were stained with an anti-phosphor-ITGB4 (Y1510) antibody. Scale bar: 100 µm. b. DAB staining intensity was quantified using ImageJ software by measuring the mean gray value. The Y-axis represents expression levels DAB signal intensity. Error bars indicate standard deviation (s.d.) (n = 4). ***$P < 0.001$, Student's t-test. c. Tumor tissues were stained with a CD45 antibody. Magnified images in the lower panel correspond to the boxed areas in the upper panel. Scale bar: 100 µm. d. CD45-positive cells were quantified (n = 3). Results presented as mean ± SEM and compared using an unpaired t-test, **$P < 0.001$.

Supplementary Table 1. Primer sequences

Primer sequences for direct sequencing (*PTPRK* exon sequencing analysis)

Target exon	Primer sequence (5' to 3')	Product size (bp)	Condition (℃)
Exon 7	Forward: GTACAGTTGTCAGATCATGCGTCTTCT	642	68
	Reverse: AGCACTCCCATGTCCTCAAATCAGTCA		
Exon 16	Forward: TGTCTCCCTGCATCTCACAGTGTCTGT	595	68
	Reverse: CCCATTCCCAGGTACACACACTTGGTT		
Exon 17	Forward: GTACCTTGCTTCAAAGACCACTGGGC	788	68
	Reverse: CACAGGGTGAAGTGGCTGAGTCAATT		
Exon 18	Forward: AGTGCAGAAGAAAAGGATCCAACCCGT	649	68
	Reverse: ATCCCTGTACAGCCAAATCTACAGCCC		
Exon 19	Forward: TCCTCGTCCATTTGTAATGCAGTGTGT	799	68
	Reverse: AACTTGCTGCAAGAAGACAGGCCTCTT		
Exon 20, 21	Forward: GCTGTCTGGTTGATGGTGGTATAAGAGT	999	68
	Reverse: GGACATAACCCGTCACTCCCTCACTT		
Exon 22	Forward: AGCTCCTAGTCCATGTTAGGCCTTCA	770	68
	Reverse: GCAGCACAAATCCTTGCAATTTAGGCC		
Exon 23, 24	Forward: CCCCCACCATTTGCATTTCCTGTGAA	1999	68
	Reverse: TCCAGGGTTAAGCAAACTTCCAGGGT		

Primer sequences for pyrosequencing (*PTPRK* mutation analysis)

Target region (amino acid change)	Primer sequence (5' to 3')	Product size (bp)	Condition (℃)
chr6:128505690 A>C (p.L350*)	Forward: TGCTCCTCCTCAGCTTCTTG	221	60
	Reverse: GGGACACCGCTGATCGTTTACGGATCTCATATTCGGTATCTGG		
	Sequence primer: AATGCTCCAACTTACAAA		
chr6:128319866 T>A (p.E893V)	Forward: GCCAGATGAGAACCACAGTGC	331	60
	Reverse: GGGACACCGCTGATCGTTTAAATCCCCTCCTTGTTGTGTTAA		
	Sequence primer: GGGTTCAAAGAGGAATAT		
chr6:128304410 G>A (p.R1122C)	Forward: TGGCTGCTACATTGTGATTGAC	144	64
	Reverse: GGGACACCGCTGATCGTTTAACATACCTCTGTCTGGACCATATT		
	Sequence primer: CAAAGCCTTAAGATCTCG		
chr6:128294824 G>A (p.T1373M)	Forward: GGTTATCTGATGGTGCAACAGTT	267	60

	Reverse: GGGACACCGCTGATCGTTTACCATAAGATGCAAGCCAGATAGTC		
	Sequence primer: GGAAGGGGAAGGCCG		
Universal primer	*GGGACACCGCTGATCGTTTA		
	*modifying 5’-biotin		

Primer sequences for pyrosequencing (*KRAS* and *BRAF* mutation analysis)

Target region	Primer sequence (5' to 3')	Product size (bp)	Condition (℃)
KRAS codon 12, 13	Forward: GGCCTGCTGAAAATGACTGAA	62	58
	Reverse: *TTAGCTGTATCGTCAAGGCACTCT		
	Sequence primer1: TGTGGTAGTTGGAGCTG		
	Sequence primer2: TGTGGTAGTTGGAGCT		
	Sequence primer3: TGGTAGTTGGAGCTGGT		
BRAF codon 600	Forward: ATGCTTGCTCTGATAGGAA	228	54
	Reverse: *GCATCTCAGGGCCAA		
	Sequence primer: GGTGATTTTGGTCTAGCTAC		
	*modifying 5’-biotin		

Primer sequences for *TP53* direct sequencing

Target exon	Primer sequence (5' to 3')	Product size (bp)	Condition (℃)
Exon5	Forward: CACTTGTGCCCTGACTTTCA	267	50
	Reverse: AACCAGCCCTGTCGTCTCT		
Exon6	Forward: AGGCCTCTGATTCCTCACTG	200	50
	Reverse: GCCACTGACAACCACCCTTA		
Exon7	Forward: AAAAGGCCTCCCCTGCTT	212	56/54/52/50
	Reverse: GTGTGCAGGGTGGCAAGT		
Exon8	Forward: TGATTTCCTTACTGCCTCTTGC	248	50
	Reverse: AAAAGTGAATCTGAGGCATAACTG		

Primer sequences for MSI analysis

Target region	Primer sequence (5' to 3')	Product size (bp)	Condition (℃)
BAT25	Forward: TCGCCTCCAAGAATGTAAGT	124	58
	Reverse: TCTGCATTTTAACTATGGCTC		
BAT26	Forward: TGACTACTTTTGACTTCAGCC	122	58

	Reverse: AACCATTCAACATTTTTAACCC		
D2S123	Forward: AAACAGGATGCCTGCCTTTA	211	60
	Reverse: GGACTTTCCACCTATGGGAC		
D5S346	Forward: ACTCACTCTAGTGATAAATCG	125	55
	Reverse: AGCAGATAAGACAGTATTACTAGTT		
D17S250	Forward: GGAAGAATCAAATAGACAAT	152	52
	Reverse: GCTGGCCATATATATATTTAAACC		

Primer sequences for pyrosequencing (Bisulfite methylation analysis of CIMP marker genes)

Gene	Primer sequence (5' to 3')	Product size (bp)	Condition (℃)
p16	Forward: GGTTGTTTTYGGTTGGTGTTTT	282	58/56/54/52
	Reverse: *ACCCTATCCCTCAAATCCTCTAAAA		
	Sequence primer: TTTTTGTTTGGAAAGAT		
MINT1	Forward: GGTTTTTTGTTAGYGTTTGTATTT	132	55
	Reverse: *ATTAATCCCTCTCCCCTCTAAACTT		
	Sequence primer: TTTAGTAAAAATTTTTTGGG		
MINT2	Forward: AGTGTTAGAAAAATGTGTTG	151	50/48/46/44
	Reverse: *CTACAATTAAACATCAATTATAT		
	Sequence primer: GAATTTTAGTATTTAAGTT		
MINT31	Forward: TGGTTTTAGTAAAGTGAGGG	117	55
	Reverse: *AACCTAATAAATCACTCAATTC		
	Sequence primer: TGGTGATGGAGGTTATT		
hMLH1	Forward: TTGATTGGTATTTAAGTTGTTTAATT	282	58/56/54/52
	Reverse: *CCAATTCTCAATCATCTCTTTAATA		
	Sequence primer: AGTTATAGTTGAAGGAAGAA		
	*modifying 5'-biotin		

Primer sequences for qRT-PCR

Target gene	Primer sequence (5' to 3')	Product size (bp)	Condition (℃)
PTRPK	Forward: ACAATTGATGGGGAGAGCAG	96	58
	Reverse: TGGCAGAGGGTATTGTGTGA		
β-actin	Forward: AGATGACCCAGATCATGTTTGAGACC	120	58
	Reverse: CGTCACCGGAGTCCATCACG		

Primer sequences for CRISPR/CAS9 genome editing validation

Primer name	Primer sequence (5' to 3')	Product size (bp)	Condition (℃)
Specific PCR primers for mutation induced genomic sequence	Forward: CCACTGTAGGATGAGTCTGGT	850	58
	Reverse: CAGCTAGCTCGATGGTACC		
Genomic DNA sequencing	Forward: TGGCTGCTACATTGTGATTGAC	406	58
	Reverse: CATAAGCAGGCTTCTAAAATGG		

Primer sequences for vector construction

Primer name	Primer sequence (5' to 3')
Primer A	CAAAAGCTTTTGGCGACCGCGAAGAAGGAAAAAG
Primer B	ACCCTCGAGAGATGATTCCAGGTACTCCAAAGCTACATCA
Primer C	CAAAGCCTTAAGATCTCGGTGTATTAATATGGTCCAGACAG
Primer D	CTGTCTGGACCATATTAATACACCGAGATCTTAAGGCTTTG

bp, base pair; MSI, microsatellite instability; CIMP, CpG island methylator phenotype; qRT-PCR, quantitative reverse transcription-PCR
Target region: chr, chromosome; A, adenine; C, cytosine; T, thymine; G, guanine
Amino acid change: L, leuicine; ＊, stop codon; E, glutamic acid; V, valine; G, glycine; R, arginine

Supplementary Table S2. Clinicopathological features of 22 depressed cancers applied to sequencing analysis.

Sample No.	Gender	Tumor location	DNA alteration in *PTPRK* (Amino acid change, Domain, Somatic status)	Gene mutation *BRAF V600E*	*TP53*	*KRAS*	*APC*	*POLE*	CIMP/MSI	Sequencing
D001	M	C	chr6:128319866 T>A (p.E893V, D1, Somatic)	N	N	P	N	Wt	CIMP-N/MSS	Exome
D002	M	A	ND	N	P	N	P	Wt	CIMP-N/MSS	Exome
D003	M	S	ND	N	N	N	N	p.A846V	CIMP-N/MSS	Exome
D004	M	S	ND	N	N	N	N	Wt	CIMP-N/MSS	Exome
D005	M	A	chr6:128505690 A>C (p.L350*, Fn3, Somatic)	N	P	N	P	p.V384L	CIMP-N/MSS	Exome
D006	M	A	chr6:128304410 G>A (p.R1122C, D1, Somatic)	N	N	P	P	Wt	CIMP-P/MSS	Exome
D007	M	A	chr6:128294824 G>A (p.T1373M, D2, Germ line)	N	N	P	P	p.R1409G	CIMP-N/MSS	Exome
D008	M	S	chr6:128304511 C>A (p.G1088V, D1, Somatic)	N	N	N	NA	NA	CIMP-N/MSS	Direct
D009	M	R	ND	N	P	N	NA	NA	CIMP-P/NA	Direct
D010	M	D	ND	N	P	P	NA	NA	CIMP-P/MSS	Direct
D011	F	T	ND	P	N	N	NA	NA	CIMP-P/MSI	Direct
D012	F	C	ND	P	N	N	NA	NA	CIMP-P/MSI	Direct
D013	M	A	ND	N	N	N	NA	NA	CIMP-N/NA	Direct
D014	M	S	ND	N	P	P	NA	NA	CIMP-N/NA	Direct
D015	F	C	ND	P	P	N	NA	NA	CIMP-P/MSI	Direct
D016	M	T	chr6:128304345 del A (NA, intron, ND)	N	P	N	NA	NA	CIMP-P/NA	Direct
D017	M	A	ND	N	N	P	NA	NA	CIMP-N/NA	Direct
D018	M	R	chr6:128303942 ins T (NA, intron, ND)	N	N	N	NA	NA	CIMP-N/NA	Direct
D019	M	S	ND	N	N	N	NA	NA	CIMP-N/NA	Direct
D020	M	T	ND	N	P	N	NA	NA	CIMP-N/NA	Direct
D021	M	D	ND	N	N	P	NA	NA	CIMP-N/NA	Direct
D022	F	S	ND	N	N	N	NA	NA	CIMP-N/NA	Direct

Gender: M, male; F, female.
Tumor location: C, cecum; A, ascending colon; T, transverse colon; D, descending colon; S, sigmoid colon; R: rectum.
DNA alteration in *PTPRK*: chr, chromosome; A, adenine; C, cytosine; T, thymine; G, guanine; ins, insertion; del, deletion; ND, not detected; NA, not applicable.
Amino acid change: L, leucine; *, stop codon; E, glutamic acid; V, valine; R, arginine; C, cysteine; T, threonine; M, methionine; G, glycine; A, alanine.
Domain: Fn3, fibronectin type-III domain; D1, protein tyrosine phosphatase D1 domain; D2, protein tyrosine phosphatase D2 domain.
Gene mutation: N, negative; P, positive; NA, not assessed.
CIMP/MSI: CIMP-P, CpG island methylator phenotype positive; CIMP-N, CpG island methylator phenotype negative; MSI, microsatellite instable; MSS, microsatellite stable; NA, not assessed.
Sequencing: Exome, all exome sequencing; Direct, direct sequencing.

Supplementary Table S3. Clinicopathological features of colorectal cancers in public database

Sample name	Tumor location	Gender	CIMP	MSI	CCS	Mutation in *PTPRK* gene	
						Site	Amino acid change (Domain of PTPRK protein structure)
TCGA-A6-2671-01	S	M	ND	MSS	3	Intron	None
TCGA-A6-3809-01	T	F	CIMP-P	MSI	2	Exon	p.V1015I (D1)
TCGA-A6-5665-01	A	F	ND	MSI	ND	Exon	p.P676Rfs＊39 (Fn3)
TCGA-A6-6141-01	C	M	ND	MSS	ND	Exon	p.T855M
TCGA-AA-3492-01	A	F	ND	MSI	2	5'UTR	None
TCGA-AA-3510-01	T	M	ND	MSS	1	Exon	p.L123F (MAM), p.S533＊ (Fn3), p.T1373M (D2)
TCGA-AA-3663-01	C	M	ND	MSI	ND	3'UTR and Exon	p.P676Rfs＊39 (Fn3)
TCGA-AA-3684-01	C	F	CIMP-P	MSS	3	Exon	p.A732P
TCGA-AA-3715-01	A	M	CIMP-P	MSI	3	Exon	p.S1071T (D1), p.L1220＊(D2)
TCGA-AA-3811-01	A	F	CIMP-N	MSI	2	3'UTR	None
TCGA-AA-3815-01	C	F	CIMP-P	MSI	2	3'UTR and Exon	p.D1237Y (D2)
TCGA-AA-3845-01	A	F	CIMP-P	MSI	2	Exon	p.N915T (D1)
TCGA-AA-3867-01	S	M	CIMP-N	MSS	3	Exon	p.F508V (Fn3)
TCGA-AA-3877-01	T	F	CIMP-P	MSI	3	Intron	None
TCGA-AA-3947-01	A	F	CIMP-P	MSI	3	Exon	p.A253T (Ig-like), p.D909Ifs＊13 (D1)
TCGA-AA-3950-01	A	F	CIMP-P	MSI	3	Exon	p.N915Kfs＊10 (D1)
TCGA-AA-3966-01	A	F	CIMP-P	MSI	3	Exon	p.L1220＊ (D2)
TCGA-AA-3977-01	S	M	CIMP-N	MSS	3	Intron and Exon	p.V425A (Fn3), p.A582V (Fn3)
TCGA-AA-A00N-01	C	M	CIMP-N	MSI	3	Intro and Exon	p.R1121W (D1)
TCGA-AA-A01R-01	A	M	CIMP-P	MSI	2	Exon	p.Q210＊ (Ig-like)
TCGA-AA-A01V-01	C	M	CIMP-P	MSS	1	Exon	p.A1196V (D2)
TCGA-AD-5900-01	A	M	ND	ND	ND	3'UTR	None
TCGA-AD-6889-01	A	M	ND	MSI	ND	3'UTR	None
TCGA-AD-6895-01	C	M	ND	MSI	ND	Exon	p.G209E (Ig-like)
TCGA-AG-3731-01	R	M	ND	ND	ND	Exon	p.F1316Y (D2)
TCGA-AG-A02N-01	R	M	ND	MSI	2	Exon	p.D689V

TCGA-AY-4071-01	ND	F	CIMP-N	MSS	1	Exon	p.E635* (Fn3)
TCGA-AY-6197-01	C	M	ND	MSI	ND	Exon	p.N282K
TCGA-AZ-6598-01	C	F	ND	MSI	ND	3'UTR and Exon	p.N915Tfs*7 (D1)
TCGA-AZ-6600-01	A	M	ND	MSS	ND	Exon	p.Q809Pfs*2
TCGA-AZ-6603-01	S	F	ND	ND	ND	Intron	None
TCGA-CA-6717-01	A	M	ND	MSS	ND	Exon	p.R331Q (Fn3)
TCGA-CK-5913-01	C	F	ND	MSI	ND	Exon	p.M1236V (D2)
TCGA-CM-4743-01	A	M	ND	MSI	ND	Exon	p.R1122H (D1)
TCGA-CM-5861-01	C	F	ND	MSI	ND	3'UTR	None
TCGA-CM-6165-01	S	M	ND	MSS	ND	Exon	p.R849H
TCGA-CM-6674-01	A	M	ND	MSI	ND	Exon	p.N915T (D1)
TCGA-DM-A1D8-01	A	F	ND	MSS	ND	Exon	p.T1322S (D2)
TCGA-EI-6507-01	R	M	ND	ND	ND	Exon	p.I1375N (D2)
TCGA-F4-6854-01	S	F	ND	MSS	ND	Exon	p.D689N
TCGA-F4-6856-01	C	M	ND	MSI	ND	Exon	p.Y1035Tfs*61 (D1)
TCGA-G4-6304-01	T	F	ND	MSI	ND	Exon	p.E437K (Fn3)
TCGA-G4-6309-01	S	F	ND	MSI	ND	Exon	N915Kfs*10 (D1)
587220	ND	ND	ND	MSI	ND	Exon	p.N915K (D1)
587228	ND	ND	ND	MSI	ND	Exon	p.R1089* (D1)
587238	ND	ND	ND	MSI	ND	Exon	p.I1079T (D1)
587332	ND	ND	ND	MSI	ND	Exon	p.E833D
587342	ND	ND	ND	MSI	ND	Exon	p.V129A (MAM), p.Q1281H (D2)
587350	ND	ND	ND	MSI	ND	Exon	p.P1423L (D2)
587376	ND	ND	ND	MSS	ND	Exon	p.F1010L (D1)
587380	ND	ND	ND	MSI	ND	Exon	p.C852Y

Tumor location: C, cecum; A, ascending colon; T, transverse colon; D, descending colon; S, sigmoid colon; RSJ, rectosigmoid junction; R, rectum.
Gender: M, male; F, female.
Invasion depth: ND, not determined.
CIMP: CIMP-P, CpG island methylator phenotype positive; CIMP-N, CpG island methylator phenotype negative; ND, not determined.
MSI: MSI, microsatellite instable; MSS, microsatellite stable; ND, not determined.
CCS: CCS, colon cancer subtype; ND, not determined.
PTPRK mutation: 5'UTR, five prime untranslated region; 3'UTR, three prime untranslated region; V, valine; I, isoleuicine; P, proline; R, arginine; fs*, frame shift; T, threonine; M, methionine; L, leucine; F, phenylalanine; S, serine; A, alanine; *, stop codon; D, asparatic acid; Y, tyrosine; N, asparagine; K, lysine; W, tryptophan; Q, glutamine; G, glycine; E, glutamic acid; H, histidine; MAM, meprin-A5 protein-receptor protein tyrosine phosphatase μ domain; Ig-like, immunoglobulin-like domain; Fn3, fibronectin type-III domain; D1, protein tyrosine phosphatase D1 domain; D2, protein tyrosine phosphatase D2 domain.

Supplementary Table S4. Mutations in *PTPRK* D1 domain and corresponding features in the structure of protein tyrosine phosphatases

Sample name	Mutation	Conserved ratio among PTPs	Motifs in PTPs	Cohort
D001	p.E893V	40%	NA	Our cohort (DC)
D006	p.R1122C	100%	NA	Our cohort (DC)
D008	p.G1088V	100%	PTP signature motif (PTP loop)	Our cohort (DC)
TCGA-AA-3947-01	p.D909Ifs*13	NA	NA	Public database
TCGA-AA-3845-01	p.N915T	90%	Phosphotyrosine recognition loop	Public database
TCGA-AA-3950-01	p.N915Kfs*10	90%	Phosphotyrosine recognition loop	Public database
TCGA-CM-6674-01	p.N915T	90%	Phosphotyrosine recognition loop	Public database
TCGA-AZ-6598-01	p.N915Tfs*7	90%	Phosphotyrosine recognition loop	Public database
TCGA-G4-6309-01	p.N915Kfs*10	90%	Phosphotyrosine recognition loop	Public database
587220	p.N915K	90%	Phosphotyrosine recognition loop	Public database
587376	p.F1010L	NA	NA	Public database
TCGA-A6-3809-01	p.V1015I	NA	NA	Public database
TCGA-F4-6856-01	p.Y1035Tfs*61	NA	NA	Public database
TCGA-AA-3715-01	p.S1071T	NA	NA	Public database
587238	p.I1079T	40%	PTP signature motif (PTP loop)	Public database
587228	p.R1089*	100%	PTP signature motif (PTP loop)	Public database
TCGA-AA-A00N-01	p.R1121W	NA	NA	Public database
TCGA-CM-4743-01	p.R1122H	100%	NA	Public database

PTPs, protein tyrosine phosphatases; NA, not applicable. E, glutamic acid; V, valine; G, glycine; R, arginine; C, cytosine; D, asparatic acid; I, isoleuicine; fs*, frame shift; N, asparagine; K, lysine; T, threonine; F, phenylalanine; L, leucine; Y, tyrosine; S, serine; *, stop codon; W, tryptophan; H, histidine; DC, depressed cancer.
